# Certain investigation on hybrid neural network method for classification of ECG signal with the suitable a FIR filter

**DOI:** 10.1038/s41598-024-65849-w

**Published:** 2024-07-02

**Authors:** Dinesh Kumar Jayaraman Rajendiran, C. Ganesh Babu, K. Priyadharsini, S. P. Karthi

**Affiliations:** 1 Department of Electronics and Communication Engineering, Sri Krishna College of Engineering and Technology, Coimbatore, Tamil Nadu India; 2 Department of Electronics and Instrumentation Engineering, Bannari Amman Institute of Technology, Sathyamangalam, Erode, Tamil Nadu India

**Keywords:** DWT-Discrete wavelet transform, ECG-Electrocardiogram, HNN-Hybrid neural network, HMLM-Hybrid machine learning models, MFIR-Modified finite impulse response, OKSA-Optimized Kogge stone adder, Computational models, Computational platforms and environments, Hardware and infrastructure, Computational biology and bioinformatics, Medical research

## Abstract

The Electrocardiogram (ECG) records are crucial for predicting heart diseases and evaluating patient’s health conditions. ECG signals provide essential peak values that reflect reliable health information. Analyzing ECG signals is a fundamental technique for computerized prediction with advancements in Very Large-Scale Integration (VLSI) technology and significantly impacts in biomedical signal processing. VLSI advancements focus on high-speed circuit functionality while minimizing power consumption and area occupancy. In ECG signal denoising, digital filters like Infinite Impulse Response (IIR) and Finite Impulse Response (FIR) are commonly used. The FIR filters are preferred for their higher-order performance and stability over IIR filters, especially in real-time applications. The Modified FIR (MFIR) blocks were reconstructed using the optimized adder-multiplier block for better noise reduction performance. The MIT-BIT database is used as reference where the noises are filtered by the MFIR based on Optimized Kogge Stone Adder (OKSA). Features are extracted and analyzed using Discrete wavelet transform (DWT) and Cross Correlation (CC). At this modern era, Hybrid methods of Machine Learning (HMLM) methods are preferred because of their combined performance which is better than non-fused methods. The accuracy of the Hybrid Neural Network (HNN) model reached 92.3%, surpassing other models such as Generalized Sequential Neural Networks (GSNN), Artificial Neural Networks (ANN), Support Vector Machine with linear kernel (SVM linear), and Support Vector Machine with Radial Basis Function kernel (SVM RBF) by margins of 3.3%, 5.3%, 23.3%, and 24.3%, respectively. While the precision of the HNN is 91.1%, it was slightly lower than GSNN and ANN but higher than both SVM linear and SVM -RBF. The HNN with various features are incorporated to improve the ECG classification. The accuracy of the HNN is switched to 95.99% when the DWT and CC are combined. Also, it improvises other parameters such as precision 93.88%, recall is 0.94, F1 score is 0.88, Kappa is 0.89, kurtosis is 1.54, skewness is 1.52 and error rate 0.076. These parameters are higher than recently developed models whose algorithms and methods accuracy is more than 90%.

## Introduction

**Figure 1:**
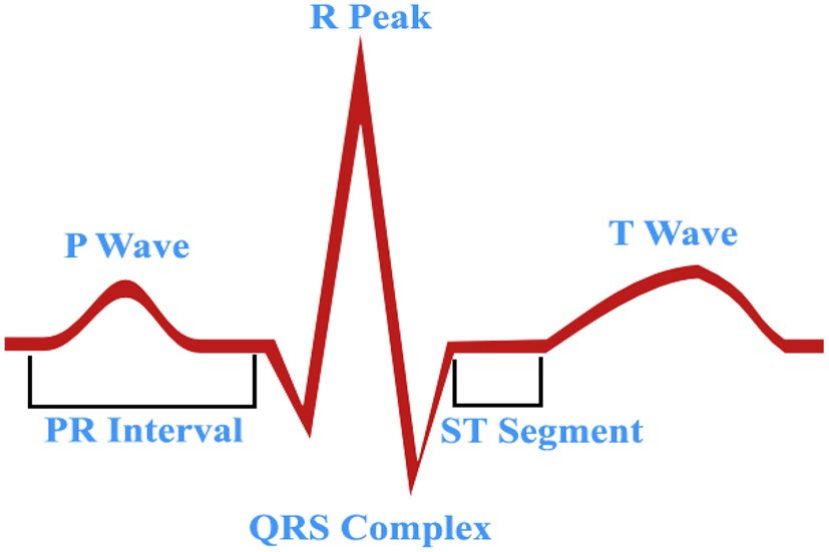
ECG Signal segments.

In general, electrocardiograms (ECGs) record heart rate and electrical activity at a given time. It is employed to record data about the functionality of the heart that arises from the depolarization of the cardiac muscles for each heartbeat^1,2^. Additionally, electrodes are positioned throughout the skin in different locations. The six peaks and valleys of an ECG signal are widely used to predict cardiac disease^3^. Figure 1 illustrates the valleys and peaks are typically identified by the symbols P, Q, R, S, T, and U. The ECG signal is a non-stationary bio-electric signal that contains critical clinical data^4^. Clinical data is often derived from measurements of the position, size, and rhythm of each heartbeat. Observations from the ECG signals include the conduction system, cardiac medication effect, presence of heart muscle cells, and functionality of implanted pacemakers^5^. To identify potentially fatal arrhythmias, trained cardiologists must carefully examine the ECG signals^6^. On the other hand, the computerized analysis of automatic classification of cardiac disorders can provide objective and diagnostic results by minimizing the time and effort of cardiologists. However, different types of noise cause harm to the information components, resulting in a wandering baseline^7^. The drifting baseline causes irregular screen patterns and it repeated throughout from the ECG Recordings^8^. The reasons for this include patient mobility, defective electrodes, electromagnetic interference, improper electrode site preparation, noise from other high-frequency electronic devices, and improper electrodes position^9^. Inappropriate clinical measures and deceptive elements can be observed by the use of noise-changing waveforms in ECG signals. In order to achieve the transparent ECG signal and improve the signal-to-noise ratio (SNR), the investigators must use denoising methods^10,11^. A median filter, sophisticated averaging, Fourier transforms, adaptive filtering, and Wavelet Transform (WT) are various methods available for ECG denoising for both linear and non-linear systems^12^. Therefore, it is advisable to modify the processing algorithm in order to improve the quality of the ECG data. ECG denoising approaches reduce noise and remove high-frequency from ECG database as non-stationary signal components. These are essential for waveform recognition; they nevertheless have certain drawbacks. Therefore, the filter must remove baseline wandering noises, which helps to generate a signal. It is error free and reconstruct the original waveform characteristic without distortion. The Empirical Mode Decomposition (EMD) has emerged as a crucial method for processing non-stationary and non-linear signals in recent times. EMD functions as a substitute for such methods. Nevertheless, certain real-time application needs are not met by this EMD^14,15^.

This paper express the methods for improvements in VLSI circuits for processing the ECG signal and explains effective computational methods for analyzing and improving. ECG signals are obtained from the publicly available Data-Base (DB) that are readily available. The data source is consisting of data for training and testing ECG signal. They are MIT-BITA, PTB, AHB-Physionet, EDB and St. Petersberg DB. These DB are collected publicly and available for the analysis of ECG–HRV variations. These DB are physiologically triggered and stimulated under the different conditions. The MIT-BIT and Physio-net ECG dataset is the source where the features are extracted. The dataset, which is utilized for feature extraction processing uses the SIMULINK workspace of MATLAB. The crucial information about ECG signals are extracted in this section and it is preprocess via the IIR and FIR filter. The suggested enhanced IIR filter coefficients are used to denoise the signals. The extracted features are fed as an input to the WT, and the classification is achieved by the neural network classifier model. Various performance metrics such as accuracy, precision, F1-measure, and recall are evaluated and compared with existing approaches. The model establishes a better trade-off compared to different methods. The paper is structured as follows: Section "Related works" offers a detailed review of the existent methodologies and models. Section "Proposed work" presents the proposed work, problem description and lays the groundwork for future improvements. By analyzing the ECG signal noise by using FIR and IIR filters, the feature extraction from the ECG signal can be enhanced. Section “Classification using neural network and hybrid tuned parameters” discuss about the result findings through the implementation of work for prediction of cardiac and classifications.

## Related works

Understanding ECG Waveforms and Their Significance Electrocardiograms (ECGs) measure the potential difference, or voltage fluctuations, between two locations in the body, revealing crucial details about the heart's electrical activity. Each waveform on ECG represents a different phase of the heart's electrical cycle. The P wave indicates the electrical activity of the atria, which initiates their contraction. Following this, the QRS complex represents ventricular depolarization, where electrical impulses spread through the ventricles, causing them to contract and pump blood. After contraction, the T wave reflects ventricular repolarization, the process by which the ventricles reset electrically for the next heartbeat. The PR interval, from the beginning of the P wave to the start of the QRS complex, measures the time required for electrical impulses to travel from the atria to the ventricles. Finally, the QT interval, spanning from the start of the QRS complex to the end of the T wave, represents the total time needed for ventricular contraction and repolarization. Understanding these components is essential for analyzing the heart's electrical function and diagnosing potential cardiac abnormalities.

### Method of decomposing—EMD’s

The existing investigation of Emphatical Mode Decomposing (EMD)-based filtering techniques is given by Bae et al.^16^. ECG signal categorization is analyzed by the researchers using different experimental strategies based on ML and AI. These studies include feature extraction, pre-processing, filtering, and classification^17^. The author^18^ covers three different methods of denoising: (i) adaptive filtering by inference extraction, (ii) adaptive filtering based on EMD, and (iii) EMD-based partial reconstruction. These innovative methods seek to reduce the noise cancellation over the ECG signal at a certain frequency (48–51 Hz) based on the fluctuating noise amplitudes. However, these strategies encapsulate the adaptive EMD improvements which were narrated in the method^18^ and it embraces the signal dependency feature. These researches subsequently demonstrate enhanced performance in reducing the wavelet noise. This improved performance is typically associated with two different requirements: (a) there are no limitations for the signal strength to be more than the noise, and (b) lower SNR’s^19–21^. Mukherjee et al., proposed a method for improving ECG signals classification based on higher-order statistics with mixed interval threshold function. This method follows the complete ensemble EMD process with adaptive noise cancellation^22^. Afterwards, the EMD approach broke down the ECG signal into the Intermediate Frequency (IMF) components group. Three distinct categories of IMFs are identified based on the frequency of noise: (i) lower frequency noise IMFs, (ii) higher frequency noisy IMFs, and (iii) noiseless relevant IMFs^23^. These approach groups are classified according to the 4th order filter by the Adaptive Derived Criterion (ADC). By combining the threshold IMFs with defined levels, the ECG signal is reconstructed. A novel ECG denoising technique based on Discrete Wavelet Transform (DWT) and EMD algorithms were analyzed in^24^ for generating the denoised ECG. This method uses the windowing methods in the EMD domain to reduce the noise generated by the initial IMF. However, using an adaptive soft thresholding technique to reduce noise cause the ECG signal (derived from data-source) fluctuates in the DWT domain as specified in^25^.

For energy conservation in the presence of noise, the ECG signal is rebuilt with a lower latency. Hence, the results in improvements of ECG classification is crucial with DWT features compared to the EMD technique. In order to process a error free ECG signal, it is necessitate stable maintenance of the QRS points^26^. Magsi et al., proposed method which improves the noise filtering performance using the EMD technique with FIR^27^. By lowering mode-mixing close to the IMF's scales supports the better grouping of ECG-HRV’s. Nonetheless, the researcher investigates and simulate an ECG filtering method that depends on lower IMF scales with higher frequency. This paper specifically highlights the inferior filtering performance with Enhanced EMD (EEMD) against the presently used EMD technique. Another noise filtering method called Weiner filtering is used to assess the filtering capabilities with EEMD. However, the model does not meet the necessary conditions^28,29^. In order to address these problems, this research focuses on modeling a system for ECG signal augmentation by noise reduction utilizing IIR and FIR filters^30–32^. To increase prediction accuracy, the ECG signal goes through feature extraction, classification, and noise reduction^33,34^. Table 1 lists the different methods of existing systems and the constraints we observed.

**Table 1:** Comparison of various existing approaches.

References	Methodology	Constraints
Praveen et al. (2024)^36^	Discusses the significance and applicability of interpretability over various healthcare applications	(1) Provides huge emphasis towards feature significance-based discussion and explanation for diverse ML approaches (2) Constraint discussion over the pros and cons of the interpretation approaches
Zhu et al., (2024)^38^	(1) Gives clear overview on various existing ML approaches (2) Also discusses the issues related to evaluation and implementation of ML approaches	The analysis does not provide ML interpreting model for ECG signals-based heart disease classification
Mishra et al. (2023)^39^	Novel deep learning approach is adopted for locating and detecting myocardial infractions	It does not include ML interpretability for myocardial infraction detection
Le et al. (2023)^35^	Provides extensive analysis on various deep learning approaches for predicting and classifying five diverse kinds of heart disease prediction with ECG signals	Gives shallow and constraint discussion over the interpretable models
Bao et al. (2021)^4^	Gives a wider analysis on various existing learning approaches in health care application	Only certain piece of work was reviewed that it concentrates on ML-applications on ECG signals based on heart disease classification
Haung et al., (2021)^14^	Gives wider explanation with the ML approach quality and Outlines the challenges encountered in other AI interpretable	Concentrates on societal impact of AI interpretable Shows constrain discussion with approaches involved in healthcare field, i.e. ECG-based heart disease classification
Jack et al. (2021)^34^	Gives detailed analysis on the constraints on pros and cons in diverse ML approaches for certain domain application and healthcare adoption Discusses the credibility and trustworthiness in ML approaches	Interprets ML approaches for ECG signals-based heart disease classification

### AI-based hybrid anomaly detection (AIHAD)

Terzi et al., proposed the AIHAD system to improve early and accurate diagnosis of coronary artery disease (CAD), especially in asymptomatic patients. AIHAD enhances diagnostic reliability by integrating various data sources beyond ECG, thus enabling quicker medical responses and reducing mortality associated with cardiovascular conditions^41^. But the CAD are highly suffered by Power line interference during the ECG examinations. Shi et al., introduced a method of hybrid Deep Net (HybDeepNet), a comprehensive method for ECG data analysis that includes hyperparameter optimization, feature extraction, and arrhythmia classification^42^. Utilizing a multilayer perceptron for tuning, a restricted Boltzmann machine for feature extraction, and a deep belief network for classification. The HybDeepNet is validated across two ECG datasets to ensure accurate predictions and efficient diagnosis. This system exemplifies the use of deep learning models such as AlexNet and LeNet to automate ECG signal classification and improve diagnostic accuracy. Multilayer Heart Attack Classification Model developed by Bhanjaa et al., as a multilayer model to enhance heart attack detection using machine learning^43^. Their approach classifies ECG signals into multiple categories to improve heartbeat detection efficiency. Furthermore, they propose an IoT-based framework with real-time notification capabilities for medical decision support and continuous patient monitoring, aiming to optimize the responsiveness and effectiveness of heart attack diagnostics. El Boujnouni et al., presents an innovative approach combining wavelet decomposition with a capsule network (CapsNet). This method classifies ECG segments and diagnose eight cardiovascular conditions. This method seeks to automate ECG analysis, reduce diagnostic errors, and expedite the diagnostic process by integrating wavelet feature extraction with CapsNet, thus pushing the frontiers of cardiovascular disease diagnosis^44^.

### AI-hybrid approaches

Combining multiple techniques like feature extraction and classification is critical for improving diagnostic performance. The cloud frameworks and notification systems are proposed to facilitate immediate medical interventions. Advanced Techniques such as Utilization of cutting-edge deep learning and signal processing techniques aims to enhance the accuracy and speed of diagnosis. This method supports the advancements in AI applications for ECG data analysis, emphasizing more precise, rapid, and automated cardiovascular disease diagnostics.

However, these methods does not ensure the suitable method to classify the ECG arrythmias. These prevalent methods are affected by the noise which cause the lower accuracy in the prediction and diagnosis of ECG’s. Hence, these models are required modification because of grow in AI prediction and usage of AI in medicals for diagnosis. This can be improved by including the filtering process with distributed methods of computing. The upcoming section describes how the proposed methods eliminate the noise generated in ECG signal during the examination and it improvise the quality of prediction with state of art of expected accuracy.

## Proposed work

### Problem statement

**Figure 2:**
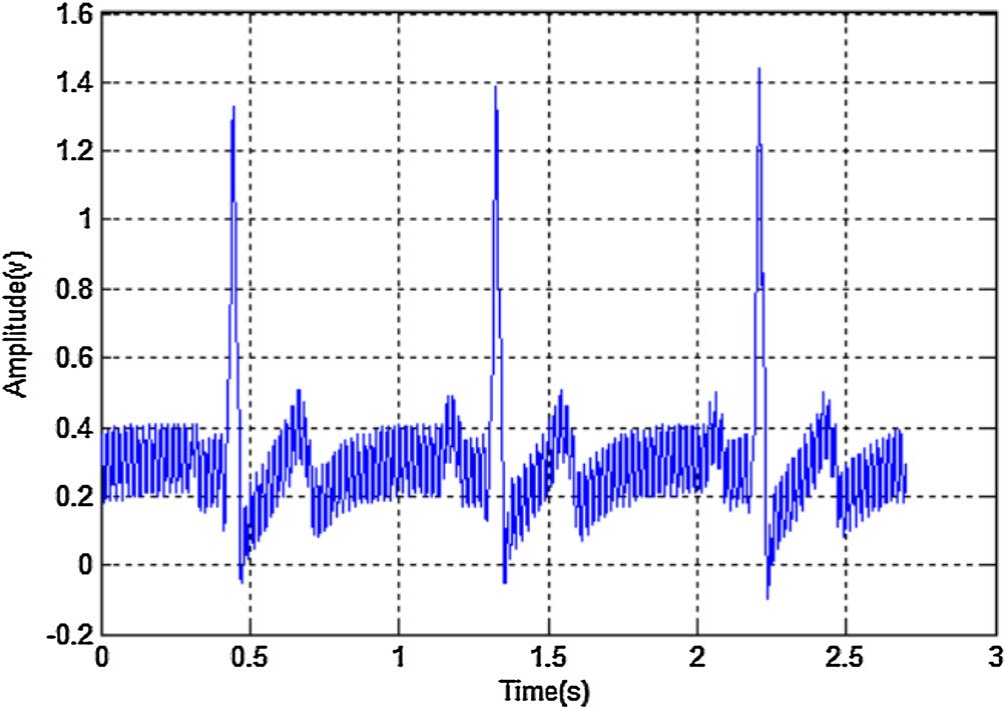
ECG signal with power frequency interference.

The problem statement and the proposed model is presented in this section. The previous section (Survey) insists that the selecting the DB and removing the noises are significant in ECG classifications. Therefore, this section focuses on the prevalent FIR filter and design restrictions. The FIR filtering architecture was constructed by adder, multiplier, delay element and the feedback network; however, several researchers suggested that there is potential for this architectural style to be efficient due the developments in VLSI methods^31–34^. In order to overcome the shortcomings of the FIR filters, this work proposed a solution to solve a number of FIR filter restrictions in addition to the predicted model's accuracy improvement. Among the traditional components of FIR filter architecture, multipliers are high power consumed component. Compared to the FIR filtering architecture, the multipliers have less efficient hardware. Superior FIR filter architecture is also dependent on adder design^40^. Designing the efficient FIR filter is depends on the hardware components prescribed early. The optimization in the hardware is achieved by a successful pipelining procedure. Using FIR filters with coefficient measures is one way to overcome these limitations in IIR filter construction. The input for the feature extraction block of classifier is extracted from IIR and IIF coefficients. Here, features are extracted using wavelet transforms (WT) in discreate way. The most influential features are supplied into the classifier model of Neural Networks (NN) as an input. A few key metrics are calculated to display the ECG Enhancement by reducing the error rate. Different methods for the ECG signal’s noise reduction are discussed in section "Related works". This research work has a more comprehensive analysis of FIR/IIR filters for low pass/high pass filters. Filtering techniques are extensively employed in signal processing and applied to various analyses of ECG signals. On the other hand, FIR/IIR filters are used in filtering techniques to enhance ECG signals^25^. Here the noise over the ECG is removed by both of these filters. Furthermore, the suggested methods eliminate a certain amount of intrinsic noise that agglutinates the ECG readings. On the other hand, contextual ECG signal filtering is found. When the intended data is still unclear and requires more execution, it is carried out. When it comes to discarding and filtering data the filtering procedure is regarded as crucial which may fall in IIR or FIR type. The denoised signals are fed into the feature extraction stage. These features are mapped as the wavelet according to frequency and time. It is found to be a useful tool for signals that are not stationary. The wavelets' resemblance to the energy spectrum and the low-frequency focus of the QRS complexes make the WT a suitable choice for denoising as shown in Fig. 2.

### A filtering approach FIR filter for adaptive and robust noise removal

The digital filters perform the reverse process (Digital to Analog) since they are converters from analog to digital. The impulse response filter design is specified by the filter coefficients. The coefficients are produced by the Linear Time Invariant (LTI) filter. The output is given by the linear coefficient’s convolution with the input sequences $Y\ast f$, as shown in Eq. (1):

1 $${\text{X}} = {\text{f}} \times {\text{Y}}$$

Here, $f$ specifies the filter impulse response, $Y$ determines the input signal, and $X$ specifies the convoluted input. The linear convolution process is expressed as in Eq. (2):

2 $${\text{X}}\left[{\text{n}}\right] = {\text{Y}}\left[{\text{n}}\right]\ast {\text{f}}\left[{\text{n}}\right]= \sum_{\text{k}}{\text{Y}}\left[{\text{k}}\right]{\text{f}}\left[{\text{n}} - {\text{k}}\right]= \sum_{\text{k}} {\text{f}}\left[{\text{k}}\right] {\text{y}}[{\text{n}} - {\text{k}}]$$

In this case, the digital input (filter), represented by y[n], the impulse response shown by f[k], and the convolution operators are denoted by “*”. It represents the impulse response of filter by providing the shifted—scaled summation. The FIR filter is a finite responsive filter and it is a type of digital filter that uses only the present and past input samples to generate the outputs. It is known as non-recursive. The FIR is performed by moving average filter by the time. With the window design technique, the low-pass filter (LPFIR) is simple and yields a superior filter output. Generally, the pass band deviation is lower than stop band variance. The window design does not allow for the independent control of these properties. In order to meet the stopband requirements, the filter in the passband must be designed without non-uniformity in the ripple (passband/stopband). As it gets further from the transition band, it gets smaller. The passband specification (φ_p_), frequency (f), stopband frequency (φ_s_), and divergence from the desired transfer function H_s_ builds the filter. The equasi-ripple FIR filter is the filter class that meets these requirements. The maximum deviation from the transfer function is decreased by this design. To lower the error, it presents a weighted approximation error between the desired and actual frequency response across the stopband and passband filters. As a result, there is a ripple in the stop and passband of the ECG- time domain representation. The weighted function of frequency response is defined by w (ω), whereas the frequency response of the filters is specified by hd (ω). The relative error magnitude can be chosen by the designer across a range of frequency bands. It is expressed as in Eq. (3):

3 $$ {\text{H}}\left( \omega \right) = e^{{ - {\text{j}}\omega \left( {{\text{M}} - 1} \right)/2}} {\text{e}}^{{{\text{j}}\left( {{\text{p}}/2} \right){\text{L}}}} {\text{H}}^{\ast} \left( \omega \right) $$

The weighted error approximation is depicted as:

4 $$\text{E}\left(\upomega \right)=\text{W}\left(\upomega \right)[{\text{H}}_{\text{d}}\left(\upomega \right)- {\text{H}}^{\ast}\left(\upomega \right)]$$

5 $$\text{E}\left(\upomega \right)=\text{W}\left(\upomega \right)[{\text{H}}_{\text{d}}\left(\upomega \right)-\text{P}\left(\upomega \right)\text{Q}\left(\upomega \right)]$$

The $\text{Q}\left(\text{w}\right)$ represents the frequency function,

6 $$\text{E}\left(\upomega \right)=\text{W}\left(\upomega \right)\text{Q}\left(\upomega \right)[{\text{H}}_{\text{d}}\left(\upomega \right)/\text{Q}\left(\upomega \right)-\text{P}(\upomega )]$$

Therefore, the approximate prediction of the coefficient set to reduce the maximal $\text{E}(\upomega )$ value (frequency bands). The approximation is expressed as in Eq. (7):

7 $$\left|\text{E}\left(\upomega \right)\right|=\text{min}[\text{max}|\text{E}\left(\upomega \right)|]$$

Using the SIMULINK Matlab tool, FIR filter coefficients are generated. The filter coefficients determine the level of filters precision and response. It is unlikely that the accurate filter requires the filter coefficients and operated by the hardware. Coefficient rounding off method is used to solve this issue^42^. Because of its ability to lower hardware use, it minimizes the hardware utilization. Performance of the filter is affected by the depreciation of filter coefficients, especially in cases where the number of tabs is considerably greater.

### Low pass series and parallel FIR filter

**Figure 3:**
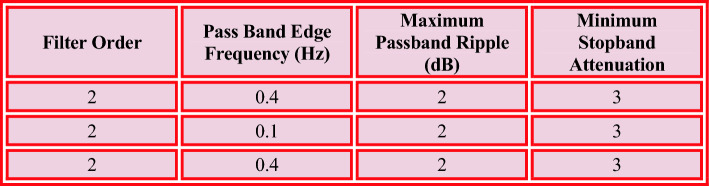
FIR parameter, IIR parameter and HPF parameter.

Single multiplier, adder, and delaying unit are required for constructing the serial FIR filter^11^. As such, it is the better choice in terms of hardware efficiency. Nevertheless, the architecture is used to implement the FIR filter, which is slower and has a lower device throughput. Similarly, the low pass parallel FIR filters analyze data in parallel. It offers higher throughput. Every adder is linked to the previous adder section output. The output terminal and adder's total are added to generate the response. The legitimate output is produced, and the delay is associated with the critical delay. Dealy plays crucial role, which is greater than or equal to the sum of the multiplier and adder delays. In this case, the adders and data in a serial fashion while remaining are connected to the Brun tree adder models^24^. When compared to the previous design, it is really beneficial by lowers the FIR filters critical delay as specified in Fig. 3.

### Analysis with IIR filter

It is a recursive filter in which the result is not depend on past inputs. It is connected to the output (linear filter). The IIR filter serves as the foundation for two distinct design strategies: (i) indirect and (ii) direct. By limiting the transfer function's pole and zero distribution, it is used to simulate IIR filters. Analog filter models preferred for indirect techniques, which modify each filter parameter in accordance with specifications. Further, the Laplace transform (LT) is used to translate the analog filters from the T-domain to the S domain by converting the frequency domain. Thus, it creates the IIR filters as the digital filter.

**Figure 4:**
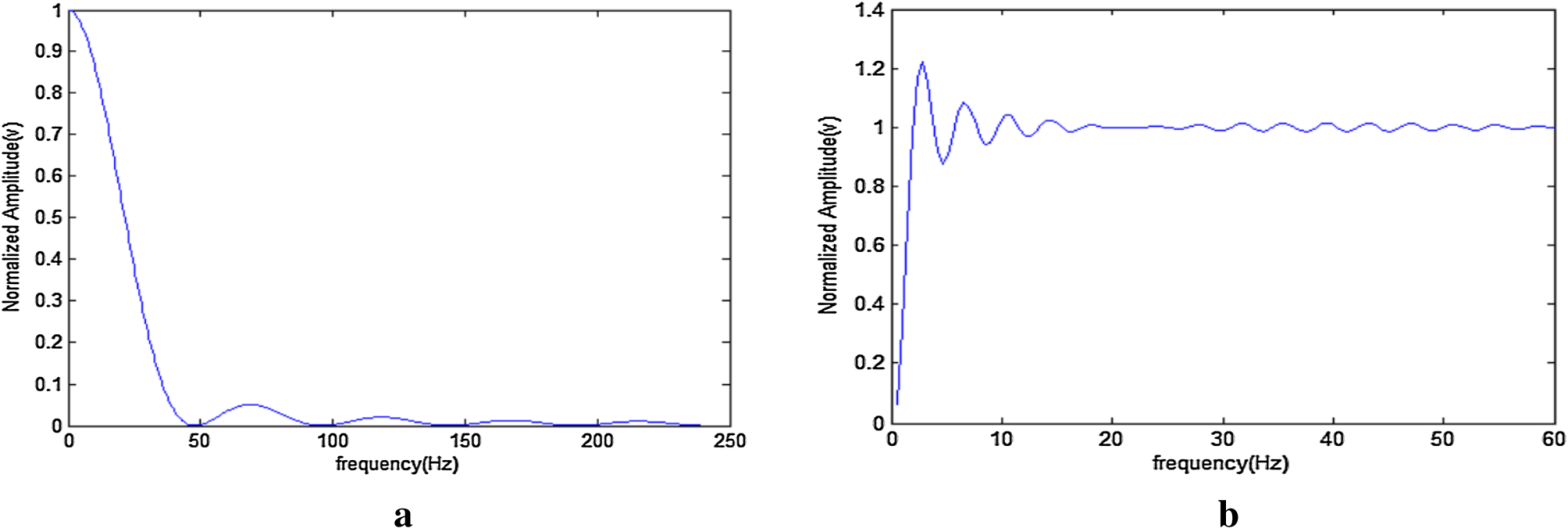
(**a**) Amplitude-frequency response of LFIR. (**b**) Amplitude-frequency response of LHFR.

The response is illustrated in Fig. 4a,b that processes digital signals. It is determined that there is a large noise influence on the obtained ECG signal. The spectrum analysis of IIR shows the variations on curve based on the real time (present) data collected during ECG acquisition. The noise is caused by baseline drift during breathing, when the frequency is set at 0–1 Hz.

The Power line Interference (PI) is another kind of noise generated during the ECG acquisition. The PI is resulting from 220 V, 50/60 Hz-AC power grid and its subsequent noise influence on the ECG signals. Two different frequency bands worth of noise signal must be avoided by building two filters. Both the low pass and high pass filters remove around 50 Hz of interference and 0 to 1 Hz of baseline drift, respectively. The Z-domain digital angular frequency is represented by the following equation, Eq. (8):

8 $$ \Omega = \frac{1}{{{\text{f}}_{{{\text{ad}}}} }}\ast{\text{ f}}_{{\text{s}}} {\ast}2\pi {\text{n}},\,\,\,\,\,{\text{n}} = 0,{ }1,2, \ldots . $$

Here, ${\text{f}}_{\text{ad}}$ specifies sampling frequency; ${\text{f}}_{\text{s}}$ specifies current frequency. Frequency ${\text{f}}_{\text{c}}=50/60$ Hz has to be avoided when ${\text{f}}_{{{\text{ad}}}} = 480{\text{ Hz}},\,\,\,\Omega_{{\text{c}}} \approx {\text{n}}\pi /5\,\,\,\left( {{\text{n}} = 0,1, \ldots ,9} \right)$. Then, n-zero is placed on $\Omega_{{\text{c}}} \approx {\text{n}}\pi /5\,\,\left( {{\text{n}} = 0,1, \ldots ,9} \right)$ to avoid the noise with maximal amplitude of $50/60\text{Hz}.$ However, another issue is the zeros in $0\text{ Hz and}\frac{50}{60}\text{Hz}$ where $(\text{1,0})$ are coordinates. The zero and pole are placed at $(\text{1,0})$ coordinated to eliminate frequency overlapping. Poles need to be located at the origin coordinate $(\text{1,0})$ to provide the transfer function and stable integer form. Generally, nine poles are placed over the unit circle to fulfill the transfer function stability. The transfer function is expressed as in Eq. (9):

9 $$ {\text{H}}\left( {{\text{j}}\Omega } \right)\frac{{\left( {{\text{e}}^{{{\text{j}}\ast 0}} - {\text{e}}^{{{\text{j}}\Omega }} } \right)\left( {{\text{e}}^{{\frac{1}{5}\pi {\text{j}} - {\text{e}}^{{{\text{j}}\pi }} }} } \right)\left( {{\text{e}}^{{\frac{2}{5}\pi {\text{j}} - {\text{e}}^{{{\text{j}}\pi }} }} } \right) \ldots \left( {{\text{e}}^{{\frac{9}{5}\pi {\text{j}} - {\text{e}}^{{{\text{j}}\pi }} }} } \right)}}{{\left( {{\text{e}}^{j0} - {\text{e}}^{j\Omega } } \right){\text{e}}^{9j\Omega } }} = \frac{{{\text{e}}^{{10\ast {\text{j}}\Omega }} - 1}}{{({\text{e}}^{{10\ast {\text{j}}\Omega )}} {\text{e}}^{{{\text{e}}j\pi }} }} $$

Here, $${\text{z}} = {\text{e}}^{{{\text{j}}\Omega }}$$. Then,

10 $$\text{H}\left(\text{z}\right)= \frac{{\text{z}}^{10}-1}{{\text{z}}^{10}-{\text{z}}^{9}}= \frac{1-{\text{z}}^{-10}}{1-{\text{z}}^{-1}}= \frac{\text{Y}(\text{N})}{\text{X}(\text{N})}$$

The filter order needs to be increased to enhance the low-pass filter characteristics. The above-given equation formula translates the low pass digital filter (Second order) and transfer function is expressed in Eq. (11):

11 $$\text{H}\left(\text{z}\right)= \frac{{\left(1-{\text{z}}^{-10}\right)}^{2}}{{\left(1-{\text{z}}^{-1}\right)}^{2}}=\frac{1-2{\text{z}}^{-10}+{\text{z}}^{-20}}{1-2{\text{z}}^{-1}+{\text{z}}^{-2}}= \frac{\text{Y}(\text{N})}{\text{X}(\text{N})}$$

Equation (11) provides the frequency-amplitude response (low pass). The algebraic sum of the filter response, or the frequency response obtained from several filters, is made by combining two linear phase filter outputs with a comparable transmission delay. By subtracting the pass filter from the low pass filter, the high-pass filter is modeled. The filter is expressed in terms of a constant lag filter, H_a_(z) = ${AZ}^{-m}$. Ha(z), H_ow_(z) has a similar DC amplification coefficient under certain ideal circumstances. When the sampling frequency is f_ad_ = 480, the low pass filter is constructed with a cut-off frequency of 2 Hz, or fc = 2 Hz. The associated angular frequency is Ωc ≈ nπ/120, where (n = 0,1,2,…,9). Considering the design of the integer filter:

12 $${\text{H}}_{\text{lp}}\left(\text{z}\right)= \frac{\text{Y}(\text{z})}{\text{X}(\text{z})}= \frac{1-{\text{z}}^{-240}}{1-{\text{z}}^{-1}}$$

Then, the subtraction of the low pass filter from the entire pass filters to model the high-pass filter. The expression is provided as in Eq. (13):

13 $${\text{H}}_{\text{hp}}\left(\text{z}\right)= \frac{\text{P}(\text{z})}{\text{X}(\text{z})}={\text{z}}^{-120}- \frac{{\text{H}}_{\text{lp}}(\text{z})}{240}$$

14 $${\text{H}}_{\text{hp}}\left(\text{z}\right)= \frac{-1+240{\text{z}}^{-121}-240{\text{z}}^{-120}+{\text{z}}^{-240}}{240-240{\text{z}}^{-1}}$$

The amplitude-frequency response (high pass filter) is provided on Eq. (14). The model design with integer filters’ transfer function is provided below.

A transfer function is designed with $2\text{ Hz}$ and $50\text{ Hz}$ low pass filter in Eq. (15):

15 $${\text{H}}_{\text{hp}}\left(\text{z}\right)= \frac{\text{P}(\text{z})}{\text{X}(\text{z})}={\text{z}}^{-120}- \frac{{\text{H}}_{\text{lp}}(\text{z})}{240}$$

Then, the differential equation is provided as Eq. (16):

16 $$\text{y}\left(\text{n}\right)=2\ast \text{y}\left(\text{n}-1\right)-\text{y}\left(\text{n}-2\right)+\text{x}\left(\text{n}\right)-2\ast \text{x}\left(\text{n}-10\right)+\text{x}(\text{n}-20)$$

Transfer function (high pass filter) is provided as Eq. (17):

17 $$\text{y}\left(\text{n}\right)=\text{y}\left(\text{n}=120\right)- \frac{(\text{y}\left(\text{n}-1\right)+\text{x}\left(\text{n}\right)-\text{y}\left(\text{n}-240\right)))}{240}$$

**Figure 5:**
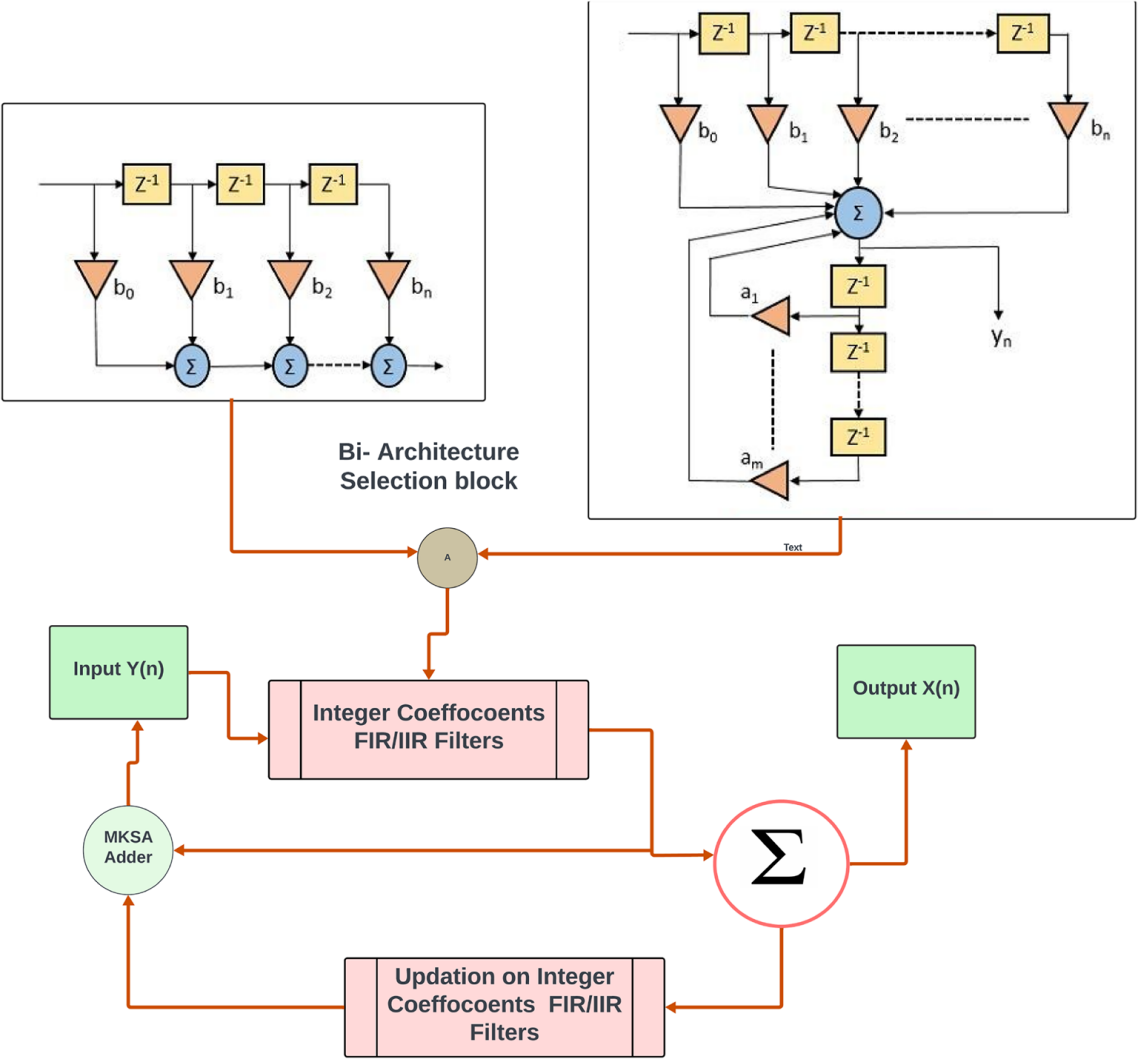
Improved FIR/IIR filter (integer coefficients) blocks with Modified Adder.

**Figure 6:**
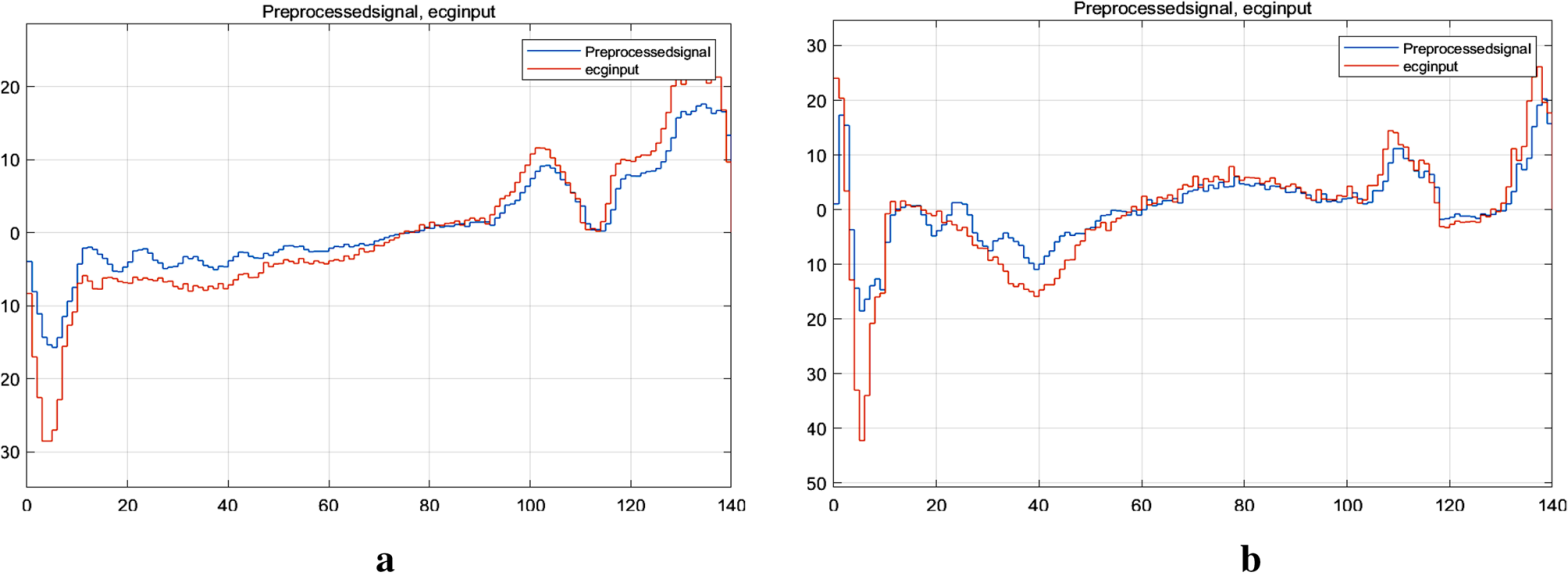
(**a**) Preprocessed ECG signal. (**b**) Preprocessed ECG signal.

Typically, ECG signals exhibit a small amount of distortion following the crucial IIR filter step (integer coefficients). As a result, the coefficients are selected in order to obtain the complete ECG signal with the significant information. It is suggested to use an improved filter coefficient to obtain the necessary data from ECG Acquisition. The structural layout of each IIR filter module, which consists of both low-pass and high-pass filters, is shown in Fig. 5. The Adder unit is replaced by Optimized Kogge stone adder (OKSA) which is defined in^40^. It has the provision to select the filter as IIR / FIR by controlling the selection block. The original signal is then subtracted from the processed signal to obtain difference signals, and ECG filters are processed using IIR integer coefficients. The compensating signal must be achieved via the filter coefficients. The signal must prevent interference while maintaining the crucial data features. The reconstruction of the waveform is the final stage. The filtering procedure extends the compensating signals which are added to achieve the final signal outcome as specified in Fig. 6. The feature from the final signal needs to extract and here DWT is used because of its better signal correlation identification and lower error rate.

### Feature extraction with wavelet transform

A wavelet, represented by x(m, n), often specifies a tiny wave that transfers the function x(t) to the time scale plane. The wavelet is more suitable for non-stationary signals. Since it provides both frequency and time information about the signal. The WT is suitable for all frequency ranges because it presents different window widths, which are broader at low frequencies and narrow at high frequencies. The WT is capable of processing and computing the features from data. The signal under different threshold scales are segregated to form the multi-scale WT’s. WT is generally represented by Eq. (18), which is a wavelet convolution ${\Psi }_{\text{m},\text{n}}$ with x(t):

18 $${\text{T}}_{\text{m},\text{n}}= \underset{-\infty }{\overset{+\infty }{\int }}\text{x}\left(\text{t}\right){\Psi }_{\text{m},\text{n}}\left(\text{t}\right)\text{dt}$$

By selecting an orthogonal wavelet basis ${\Psi }_{\text{m},\text{n}}\left(\text{t}\right)$ specifies the reconstructed original signal. The signal's approximation coefficient is expressed as Eq. (19):

19 $$ {\text{S}}_{{{\text{m}},{\text{n}}}} = { }\mathop \smallint \limits_{ - \infty }^{ + \infty } \left( {{\text{at}}} \right)\emptyset_{{{\text{m}},{\text{n}}}} \left( {\text{t}} \right){\text{dt}} $$

where $\text{M}$ and $\text{N}$ specify signal scaling and location. The discrete signal approximation is expressed as Eq. (20):

20 $${{\text{x}}_{0}}({\text{t}})={{\text{x}}_{\text{M}}}  {\text{(t)}}\sum_{{\text{m}}=1}^{\text{M}}{{\text{d}}_{\text{m}}} {\text{(t)}}$$

where, $\emptyset_{{{\text{M}},{\text{n}}}}$ specifies signal approximation (mean) at ‘${\text{m}}$’ scale is expressed as Eq. (21):

21 $$ {\text{x}}_{{\text{M}}} \left( {\text{t}} \right) = {\text{S}}_{{{\text{M}},{\text{n}}}} \emptyset_{{{\text{M}},{\text{n}}}} { }\left( {\text{t}} \right) $$

The signal approximation is related to sale ‘${\text{m}}$’ for the finite-length signal is expressed as Eq. (22):

22 $${\text{d}}_{\text{m}}\left(\text{t}\right)= \sum_{\text{n}=0}^{\text{M}-\text{m}}{\text{T}}_{\text{m},\text{n}}{\Psi }_{\text{m},\text{n}} (\text{t})$$

The signal approximation at a certain scale is the integration of approximation at the lower scale. It is expressed as Eq. (23):

23 $${\text{x}}_{\text{m}}\left(\text{t}\right)={\text{x}}_{\text{m}-1}\left(\text{t}\right)-{\text{d}}_{\text{m}} (\text{t})$$

The original signal is processed by the both low-pass and high-pass filters in the WT of multi-resolution. It obtains precise and approximate signal coefficients at different frequency constraints. The low-scale and approximations are high-scale and low-frequency signal components, whereas the details are high-frequency components. The analysis focused on the various frequency bands where the signal is broken down inter mediate coefficients and approximations. There is no uniform method for selecting a particular wavelet function; but the initial wavelet evaluation in the wavelet transforms is a crucial responsibility. Depending on the kind of signal that needs to be identified. Similar, to a wavelet function the signal is often selected. Because QRS complexes are concentrated at low frequencies and wavelets resemble the energy spectrum, the wavelet transform is a suitable tool for denoising in particular.

## Classification using neural network and hybrid tuned parameters

Numerous neurons are connected to transfer and receive data simultaneously in a Hybrid neural network (HNN). A weight is assigned to each neuron in the network. It shows the status of the network during learning, and each neuron's weight needs to be adjusted and updated. Every neuron's predicted model is fully connected to hidden layers that are used to classify the ECG data and extract characteristics. To reduce the number of features and speed up processing, the generalized sparse network model is used. The HNN is implemented in SIMULINK models. Various descriptive characteristics and statistics are displayed by the feature extraction method, which uses the pre process from the ECG signals. Based on the feature vectors, WT used to extract the features and the network model is trained to categorize ECG signals. The analysis stage is used to preserve the model efficiency in the outcome. The module defines the design for prediction using the HNN model. The neurons demonstrate the multi-stage neuron stages. The sources (features) ${\text{B}}_{1},\dots ,{\text{B}}_{\text{n}}$ is considered as unidirectional and yields neurons sign stream. The neuron output is provided in Eq. (24):

24 $${\text{O}}={\text{f (network)}}=\text{ f}\left(\sum_{\text{i}=1}^{\text{n}}{{\text{A}}_{\text{i}}}{{\text{B}}_{\text{i}}}\right)$$

${{\text{A}}_{\text{i}}}{{\text{B}}_{\text{i}}}$ specifies the weighted vector, and the capacity is specified as ${f}_{(Network)}$ the network. The information vectors and weight depict the variable network as scalar consequences. It is expressed in Eq. (25):

25 $${\text{network}}={{\text{A}}^{\text{T}}}{\text{B}}={{\text{A}}_{1}}{{\text{B}}_{1}}+{{\text{A}}_{2}}{{\text{B}}_{2}}+\dots +{{\text{A}}_{\text{n}}}{{\text{B}}_{\text{n}}}$$

where T specifies the matrix transposition of A. The value of O is represented as in Eq. (26):

26 $$\text{O}=\text{f}\left(\text{network}\right)= \left\{\begin{array}{cc}1& \text{if }{\text{A}}^{\text{T}}\text{x}\ge\uptheta \\ 0& \text{otherwise}\end{array}\right.$$

Here, the range is specified by the limit and the linear threshold unit is expressed as node type. The neuron models’ inner activity is expressed as Eq. (27):

27 $${\text{v}}_{\text{k}}={ \sum_{\text{i}=1}^{\text{p}}}{{\text{A}}_{\text{ki}}}{{\text{B}}_{\text{i}}}$$

The result of v_k_'s activation function is the neuron output represented by y_k_ It is crucial to reduce errors within the assessed ECG class. The output (expected) with the original output value is used to assess the network's performance. The suggested method is quicker, but it requires more data to proceed. It is similar to back-propagation neural networks, in which errors are corrected and forwarded. With the stated X = a, the classifier's relapse is unavoidable due to its desire. It provides the scalar value that the input vector is represented by b/a. Let scalar arbitrary variable Y and vector irregular variable capacity be represented by f(a,b) is helpful to assess the random estimation. The relapse is provided as Y, and the restrictive mean is expressed as in Eq. (28):

28 $${\text{E}}\left[ {\frac{{\text{Y}}}{{\text{X}}}} \right] = \int_\infty ^\infty  {{\text{Y}},{\text{f}},\left( {\frac{{\text{b}}}{{\text{a}}}} \right){\text{dy}}}  = \frac{{\int_\infty ^\infty  {{\text{Y}},{\text{f}}({\text{a}},{\text{Y}}){\text{ dy}}} }}{{\int_\infty ^\infty  {{\text{f }}({\text{a}},{\text{ Y}}){\text{ dy}}} }}$$

Here, X and Y specify the ex-squeezed factors with specific parameters. The essential structure among $\text{a }$and $b$ is a non-parametric estimation with no prior information.

### Loss functions

Define abbreviations and acronyms the first time they are used in the text, even after they have been defined in the Eq. (29). Assume N pair of training sample dataset:

29 $$\text{D}=\{({\text{a}}_{\text{s}}, {\text{b}}_{\text{s}})|\text{s}=1,\dots ,\text{N}\}$$

where, ${\text{a}}_{\text{s}}\in {\text{R}}^{\text{n}}$ specifies the input vector and ${\text{b}}_{\text{s}}\in \text{R}$ specifies the output of ${\text{a}}_{\text{s}}.$ The target is to compute the function with € deviation of appropriate outcome for the entire training dataset and the relationship among ${\text{a}}_{\text{s}}$ and ${\text{b}}_{\text{s}}.$ It is based on converting the sample training set to high dimensional kernel feature space that relies on $\varphi (.):{\text{R}}^{{\text{n}}} \to {\text{R}}^{{\text{m}}}$, and the linear modelling is provided as in Eq. (30):

30 $$ {\text{f}}\left( {\text{a}} \right) = {\text{w}}^{{\text{T}}} \varphi \left( {\text{a}} \right) + {\text{c}} $$

where $\text{w}\in {\text{R}}^{\text{m}}$ specifies the weighted vector, C specifies the threshold parameter, and W represents minimal Euclidean like Eq. (31):

31 $$\text{w}={\left|\left|\text{w}\right|\right|}_{2}^{2}$$

The pair for precision representation is given in Eq. (31), reducing the error during desired and predicted output. To minimize the error function and expressed as Eq. (32):

32 $$\text{L}{\left({\text{e}}_{\text{s}}\right)}_{\upepsilon }=\text{L}{\left({\text{b}}_{\text{s}}-\text{f}\left({\text{a}}_{\text{s}}\right)\right)}_{\upepsilon }$$

The proposed neural network optimization problem is shown below:

33 $$\text{J}\left(\text{w},\text{c}\right)= \frac{1}{2}{\left|\left|\text{w}\right|\right|}_{2}^{2}+\text{P }\sum_{\text{s}=1}^{\text{N}}\text{L}{\left({\text{b}}_{\text{s}}-\text{f}\left({\text{a}}_{\text{s}}\right)\right)}_{\upepsilon }$$

Here, ${\text{P}} \in {\text{R}}^{ + }$ represents user-defined parameters. The amount of noise over the training samples is provided, not included in the output. Therefore, the loss function based on the optimization approach is sparse to attain the solution. It is supplied in Eq. (34):

34 $${\left|{\text{b}}_{\text{s}}-\text{f}\left({\text{a}}_{\text{s}}\right)\right|}_{\in } = \left\{\begin{array}{cc}0& \left|{\text{b}}_{\text{s}}-\text{f}\left({\text{a}}_{\text{s}}\right)\right|<\epsilon \\ \left|{\text{b}}_{\text{s}}-\text{f}\left({\text{a}}_{\text{s}}\right)\right|-\upepsilon & \text{else}\end{array}\right.$$

From the statistical analysis, the loss function is measured to be optimal. Based on the error distribution, the insensitive loss function is provided as Eq. (35):

35 $$\text{L}{\left({\text{e}}_{\text{s}}\right)}_{\text{e}}={\left({\text{e}}_{\text{s}}\right)}_{\text{e}}^{2}$$

Here, ${\left({\text{e}}_{\text{s}}\right)}_{\text{e}}^{2}$ specifies the continuous differential function. With the integration of Eq. (34) and Eq. (35), the network model is provided as Eq. (36):

36 $$\text{w }\in {\text{R}}^{\text{m}},\text{ b}{\text{R}}^{\text{J}\left(\text{w},\text{c}\right)}= \frac{1}{2}{\text{w}}_{2}^{2}+\text{P}{\sum_{\text{s}=1}^{\text{N}}{\left[\left({\text{b}}_{\text{s}}-\text{f}\left({\text{a}}_{\text{s}}\right)\right)\right]}_{\upepsilon }}^{2}$$

37 $$ = \left\{ {\begin{array}{*{20}c} {{\text{b}}_{{\text{s}}} - {\text{w}}^{{\text{T}}} \varphi \left( {{\text{a}}_{{\text{s}}} } \right) - {\text{c}} \le \varepsilon + \upxi_{{\text{s}}} } \\ { - {\text{y}}_{{\text{s}}} + {\text{w}}^{{\text{T}}} \varphi \left( {{\text{a}}_{{\text{s}}} } \right) + {\text{c}} \le \varepsilon + \upxi_{{\text{s}}} } \\ {\upxi_{{\text{s}}} ,{ }\upxi_{{\text{s}}}{\prime} \ge 0,\,\,{\text{s }} \in \left\{ {1, \ldots ,{\text{N}}} \right\}} \\ \end{array} } \right. $$

Here, $\upxi_{{\text{s}}} , \upxi_{{\text{s}}}{\prime}$ represents slack variable utilized for negative and positive deviation. To compute primal objective, the linear regression is multiplied with a non-negative multiplier for every sample set.

38 $$ {\text{w}} \in {\text{R}}^{{\text{m}}} ,\,\,{\text{b}} \in {\text{R}}^{{{\text{j}}\left( {{\text{w}},{\text{c}},\upalpha_{{\text{s}}} ,{ }\upalpha_{{\text{s}}}^{{^{\prime} }} ,\upgamma_{{\text{s}}} ,\upgamma_{{\text{s}}}{\prime} ,\upxi_{{\text{s}}} ,\upxi_{{\text{s}}}{\prime} } \right)}} = {\text{w}},{\text{c}},\upalpha_{{\text{s}}} ,\upalpha_{{\text{s}}}^{{^{\prime} }} ,\upgamma_{{\text{s}}} ,\upgamma_{{\text{s}}}{\prime} ,\upxi_{{\text{s}}} ,\upxi_{{\text{s}}}{\prime} \ge 0{ }\,\,\,{\text{s}} \in \left\{ {1, \ldots ,{\text{N}}} \right\} $$

39 $$ \frac{1}{2}{\text{w}}^{{\text{T}}} {\text{w}} + {\text{P}}\mathop \sum \limits_{{{\text{s}} = 1}}^{{\text{N}}} \left[ {\left( {\upxi_{{\text{s}}} } \right)^{2} + \left( {\upxi_{{\text{s}}}{\prime} } \right)^{2} } \right] - \mathop \sum \limits_{{{\text{s}} = 1}}^{{\text{N}}} \upalpha_{{\text{s}}} \left( {\varepsilon + \upxi_{{\text{s}}} - b_{{\text{s}}} + {\text{w}}^{{\text{T}}} \varphi \left( {{\text{a}}_{{\text{s}}} } \right) + {\text{c}}} \right) - \mathop \sum \limits_{{{\text{s}} = 1}}^{{\text{N}}} \upalpha_{{\text{s}}}{\prime} \left( {\varepsilon - \upxi_{{\text{s}}}{\prime} - {\text{b}}_{{\text{s}}} + {\text{w}}^{{\text{T}}} \varphi \left( {{\text{a}}_{{\text{s}}} } \right) - {\text{c}}} \right) - \mathop \sum \limits_{{{\text{s}} = 1}}^{{\text{N}}} \left( {\upgamma_{{\text{s}}} \upxi_{{\text{s}}} + \upgamma_{{\text{s}}}{\prime} \upxi_{{\text{s}}}{\prime} } \right) $$

Here, $\upalpha_{{\text{s}}} , \upalpha_{{\text{s}}}{\prime} ,\upgamma_{{\text{s}}} , \gamma_{{\text{s}}}{\prime}$ represents Lagrange multipliers. To attain optimal solution, the primal variable is vanished. Therefore, the partial derivation is equal to zero.

40 $$ \frac{{\delta {\text{J}}}}{{\delta {\text{c}}}} = \mathop \sum \limits_{{{\text{s}} = 1}}^{{\text{N}}} \left( {\upalpha_{{\text{s}}}{\prime} - \upalpha_{{\text{s}}} } \right) = 0 $$

41 $$ \nabla_{{\text{w}}} {\text{J}} = {\text{w}} - \mathop \sum \limits_{{{\text{s}} = 1}}^{{\text{N}}} (\upalpha_{{\text{s}}} - \upalpha_{{\text{s}}}{\prime} )\varphi { }({\text{a}}_{{\text{s}}} ) = 0 $$

42 $$ \frac{{\delta {\text{J}}}}{{\delta \upxi_{{\text{s}}} }} = {\text{P}}\left( {2\upxi_{{\text{s}}} } \right) - \upalpha_{{\text{s}}} - \gamma_{{\text{s}}} = 0 $$

43 $$ \frac{{\delta {\text{J}}}}{{\delta \upxi_{{\text{s}}} }} = {\text{P}}\left( {2\upxi_{{\text{s}}}{\prime} } \right) - \upalpha_{{\text{s}}}{\prime} - \gamma_{{\text{s}}}{\prime} = 0 $$

Substitute Eqs. (42) and (43) in Eq. (40), the model handles optimization problem:

44 $$ \mathop {\max }\limits_{{ \propto \in {\text{R}}^{{\text{N}}} }} {\text{J}}\left( {{\upalpha }_{{\text{s}}} {\upalpha }_{{\text{s}}}{\prime} } \right) = - \frac{1}{2}\mathop \sum \limits_{{{\text{s}} = 1}}^{{\text{N}}} \mathop \sum \limits_{\gamma = 1}^{{\text{N}}} \left( {\upalpha_{{\text{s}}} - \upalpha_{{\text{s}}}{\prime} } \right) - \varepsilon \mathop \sum \limits_{{{\text{s}} = 1}}^{{\text{N}}} \left( {\upalpha_{{\text{s}}} - \upalpha_{{\text{s}}}{\prime} } \right) + { }\mathop \sum \limits_{{{\text{s}} = 1}}^{{\text{N}}} {\text{b}}_{{\text{s}}} \left( {\upalpha_{{\text{s}}} - \upalpha_{{\text{s}}}{\prime} } \right) - { }\frac{1}{{2{\text{P}}}}\mathop \sum \limits_{{{\text{s}} = 1}}^{{\text{N}}} \left[ {\left( {\upalpha_{{\text{s}}} } \right)^{2} + \left( {\upalpha_{{\text{s}}}^{{{^{\prime}}2}} } \right)} \right] $$

45 $$ \mathop \sum \limits_{{{\text{s}} = 0}}^{{\text{N}}} \left( {\upalpha_{{\text{s}}}{\prime} - \upalpha_{{\text{s}}} } \right) = 0\,\,\,{\text{and}}\,\,\,\upalpha_{{\text{s}}}{\prime} \upalpha_{{\text{s}}} \varepsilon \left[ {0,\infty } \right] $$

where K specifies kernel matrix, the kernel function $\text{K}({\text{a}}_{\text{s}}, {\text{a}}_{\text{r}})$ represents the product of two samples, $\varphi \left( {{\text{a}}_{{\text{s}}} } \right)$ and $\varphi \left( {{\text{a}}_{{\text{r}}} } \right)$.

46 $$ {\text{K}} = \left[ {{\text{K}}\left( {{\text{a}}_{{\text{s}}} ,{\text{ a}}_{{\text{r}}} } \right)]_{{{\text{s}},{\text{r}}}} = \left[ {\varphi^{{\text{T}}} \left( {{\text{a}}_{{\text{s}}} } \right).{ }\varphi \left( {{\text{a}}_{{\text{r}}} } \right)_{{{\text{s}},{\text{r}}}} } \right]} \right] $$

The dual optimization problem handles quadratic problems whose outcomes are unique and minima. The optimal model and decision function of the test set samples are provided as in Eq. (47):

47 $$ {\text{w}} = \mathop \sum \limits_{{{\text{a}}_{{\text{s}}} \in {\text{DV}}}} \left( {\upalpha_{{\text{s}}} - \upalpha_{{\text{s}}}{\prime} } \right)\varphi \left( {{\text{a}}_{{\text{s}}} } \right) $$

48 $$ {\text{f}}\left( {\text{a}} \right) = \mathop \sum \limits_{{{\text{a}}_{{\text{s}}} \in {\text{SV}}}} \left( {\upalpha_{{\text{s}}} - \upalpha_{{\text{s}}}{\prime} } \right){\text{k}}\left( {{\text{a}}_{{\text{s}}} ,{\text{ a}}} \right) + {\text{c}} $$

From Eq. (48), the operation is evaluated using the input space with kernel function with training set sample to high dimensional space. Here, SV represents the training set samples $\upalpha_{{\text{s}}} - \upalpha_{{\text{s}}}{\prime} \ne 0$ while considering $\text{f}(\text{a})$, w does not need evaluation. It diminishes the computational time, and the model intends to resolve the issue.

## Hybrid numerical architecture and results and discussions

The HNN is the method of classifying the ECG signal to improve the accuracy and it ensures the minimum error rate. The HNN architecture is built on 4 different blocks, namely (i) ECG acquisition, (ii) Preprocessing, (iii) Hybrid model, and (iv) The decision maker. The stage (i) is the fundamental steps to acquire the ECG signals. The ECG system represents the Heart Rate Variability (HRV) with wavering and vary for individuals. Therefore, the 2-lead based physical signals are mapped with ECG signals which are collected from the subject body. The entire process is to detect and distinguish the HRVs. Here the classifier is to justify the harmony variation of ECG and identify the probability of phenomenon with suitable combinations. The HNN is described to analyses the ECG with the feature extractions. The feature extraction methods are briefed in^37,38^. However, the feature may not have the expected accuracy level and failed to the recognition. Hence the different feature extraction methods are implemented to the well examined dataset. There hybrid manner brings the advantage of improved accuracy. Although different models are used to extract the features, it will be combined together to form the hybrid data source to take decision. The weightage is given to the model as true (1) and false (0) based on the probability and error rate. The features obtained from the different group of subjects with minimum size are resembled to identify the prior values of probability as binary representation. The hybrid model receives the ECG data base obtained by 2-LEAD with 400 Hz and having the maximum records of 2034. Table 2 interpret the hybrid model performance for the 4 different series. The series are grouped based on the invigorating of the maximize the performance. Series_1 represents the boosted signals and series_2 represents the reassured ECG with the highest probability of true. The residue series_3 & series_4 epitomize the methodology on theoretical and expected behavior of the model irrespective of the model parameters and performance consideration.

**Table 2:** HNN model predictions.

Possible methods of interactions	Model 1	Model 2	Hybrid model
Series_1	True classification	False classification	True classifier
Series_2	False classification	True classification	True classifier
Series_3	True classification	False classification	True classifier
Series_4	False classification	False classification	False classifier

This series are considered for all the possible amalgamation of the datasets mitigate to the two models. In the case of model failure, it pushes the entire model performance to inaccurate. Here, using the hybrid model secures the better system performance, where the false classification of the one model can be overshadowed by another true model. Therefore, the hindrance of model failure and performance laid down can be eliminated. However, on the case of unexpected failure of both model classification causes the entire collapse of the model’s performance and evaluation. Table 3, list the data base (DB) used in this research work. There are 5 known databases are used here. There are MIT-BITA, PTB, AHB-Physionet, EDB and St. Petersberg DB. These DB are collected publicly and available for the analysis of ECG–HRV variations. These DB are physiologically triggered and stimulated under the different conditions. Thes DB examine the various physio activity with the diverge activities which supports the mutated ECG recording available for the study and analysis. The MIT-BITA, public DB used to compare with the contemporary research methods. The data sources are extracted from the both gender with the age group of 6–85 years. All the databases are acquisition in the frequency band of 100–500 Hz. Here, all the utilized DB are developed by the different biometric systems and emphasized in various records. The wavelets features are extracted from MIT-BITA DB, where it ensures the maximize the pattern in both time and frequency domain with the wavelet’s representation. The fiducial features are in time series and finds the crucial points of on and offset points. The fiducial feature from PTB encompasses 19 different feature variables. The EDB, St petersberg DB, AHA-Physionet and PTB are other DB used here for analysis. The proposed DB is the modified version of MIT-BIT where the sampling frequency of 360 with 11-bit resolution of 10 mV range is used. In total 1034 records are generated by the 10 LEAD combinations. The features are wavelets and fiducial stats is used here.

**Table 3:** Various data source available for ECG signals and features.

Data source	Sampling frequency	Lead	Records	Age group	Extracted features
MIT-BITA	360	2	980	21–85	Wavelets, stats
PTB	257	4	1081	NA	Correlations
AHA—Physionet	249	4	1240	NA	Fiducial, cross correlations
EDB	249	8	800	29–83	Wavelets, stats
St Petersburg DB	258	12	705	18–81	Wavelets, stats
Proposed-MDB	360	2	1034	6–86	Wavelets, stats

The subsequent section of HNN architecture is (ii) Preprocessing, where the noises present in the ECG database and noised raised by the artifact are removed in this stage. The pre-processing stages focus on the removal of white noise which may generated by the electrical activity of the heart. This stage has the sub division as FIR filter and R-detection. The FIR filter design is explained in section "Proposed work" and it is represented by the ECG of pass band filter where the frequency of the filter can be divided as sub sections. The process of the sub section is in the range of 1–50 Hz, which reduces the interfaces built by the ECG signal. Regardless of the different sampling frequency, the HNN is selected to form the sampling and non-sampling signals which could generate the independent response of each model. Therefore, the signal overlapping is reduced and the input parameters are filtered. The R detection is performed after the out-linear removal. The R detection is most successful one on identifying the HRV and interval of less than 400 mS is filtered. It is mapped to high bpm of the heart nearly 100–120, which is practically not occurs. The Hybrid model (iii) is the next section in which the two different models are combined together. In this its important to maintain the classification models are not of the same type. So that it supports the accurate classification on the verdict false and true classification.

**Figure 7:**
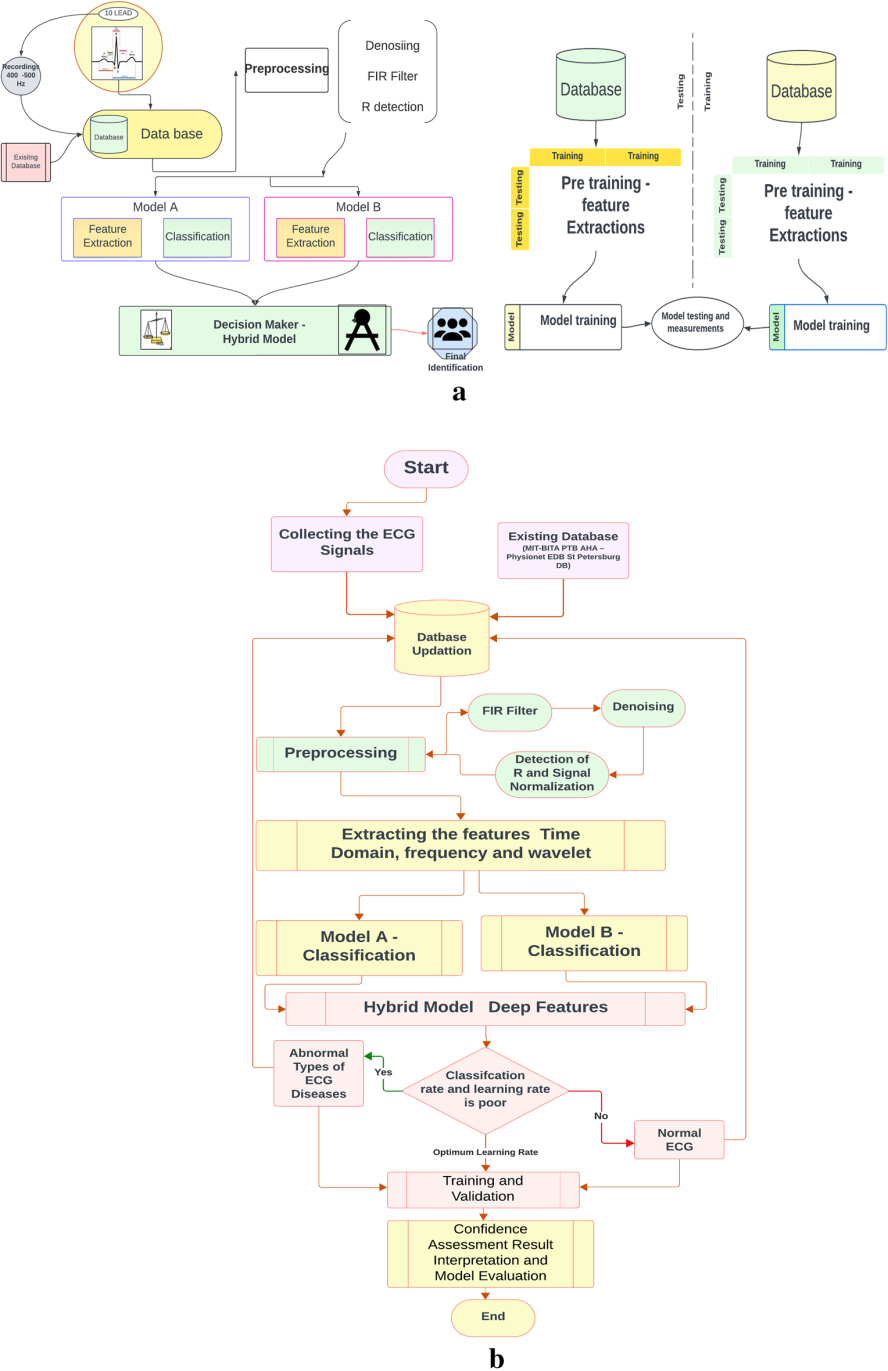
(**a**) Proposed hybrid model of decision making based on training and testing provided. (**b**) Process flow of the proposed model for ECG classifications—accurate decisions based on model interpretation and evaluation.

The embarked model improves the high accuracy on the classification. However, for the case of both model failure results the loss of the model performance and cause the unfit model on ECG classification. The practically observed ECG signals are clustered into the small oscillated signals with the variations on low (to) and high (fro) movement. The Distance based spatial clustering method (DBSCM) is used to group the beats. The group of beats are identified as the features of oscillating and the HR is invalid if the features are fall in sparse point region. However, the features in DBSCM are segregated by the oscillation and the distance between the feature point and point of reference. The distance ae measured by the statistical parameters such as mean, std. deviation and maximum distances. The points “i” of DBSC at one point is covered by the other points “j” and all the features are covered by the maximum number of feature representations. This method is named as Concentration-Based Clusters Reaches (CBCR). After the CBCR the normalized correlation of cross method is performed on the distance where HRV is occurred. Through this process CBCR in DBSC clears the HRV large deviation and discarded as noises. Figure 7a illustrates the blocks presents in the proposed model and Fig. 7b shows the proposed flow for ECG Classification based on the features extraction mentioned in the previous section. Also, it is defined by the hybrid blocks of two distinct models with better optimized features and corresponding vector values.

The Principal Component Analysis (PCA) is used to optimize the CBCR and discriminate the larger data variations with respect to the axis of representation. The Auto correlation is used for the feature extraction where the longer duration of ECG is considered. It means that, distance between the actual signal and relocated signal are obtained by the Sum of the products (SOP) through the skewed patterns. The statistical features are extracted by the monotonous signals, obtained by the FIR based band pass filter. As mentioned in^37^ all the 11 parameters are considered for the feature representations. The final process of the HNN is decision maker (DM). The DM follows the Random Forest (RF) method for classification and the decision is taken by the probability of higher value obtained from the models connected. The RF methods ensembles the multi-level classifications. For the DM, the Decision Tree (DT) classification is used to predict the HRV. However, the DT has the significant role than the RF in DM. Since the parental and root nodes of DT are obtained by different test performed at structural end point variations. This is represented by the pre trained function at the leaf node with pre space dense clusters as specified in DBSC. Hence the DT at the end points parent and child root nodes ensures the single level classifications. Figure 7 illustrates the proposed model of the HNN as per the steps explained above. In the decision maker.

The experimental designs are performed at the different combinations. The experimental space is divided with the following sections, (i) train the model with the different set of the models which are familiar in ML model. (ii) The proposed method FIR based filtered Database (PDB) with the various features, (iii) experiment evaluation on same training and testing on different features, (iv) Design space for analysis of Hybrid model (HNN) features. Table 4 shows the comparative experiment results of the different methods, such as GSNN, ANN, SVM and HNN. Here it is observed that Accuracy of HNN is 92.3% and the recall is 98.5% with the lowest error rate of 0.0761. Metrics including accuracy, precision, specificity, recall, and error rate are calculated based on the classifier output and numerous literature analyses. The simulation is carried out using SIMULINK, and the experimental results show that the performance outperforms a number of alternative methods. Below is a discussion of each metric's definition and expression. For every test fold in the dataset, the results are acquired, i.e. True Positive (TP), True Negative (TN), False Positive (FP) and False Negative (FN). TP: heartbeats are identified correctly; FN: beats inaccurately identified; TN: beats correctly identified as negative.

**Table 4:** A parametric methods and the results obtained.

Methods	Accuracy (in %)	Precision (in %)	Recall (in %)	F1-score (in %)	Error rate
GSNN	89	90	89	89	0.1568
ANN	87	81	68	69	0.2546
SVM (linear)	69	70	59	60	0.3564
SVM (RBF)	68	70	60	65	0.1856
HNN	92.3	91.1	92.5	90.6	0.0761

49 $$\text{Precision}=\frac{\text{TP}}{\text{TP}+\text{FN}}$$

50 $$\text{Recall}= \frac{\text{TP}}{\text{TP}+\text{FN}}$$

51 $$\text{F}1= \frac{2\ast \text{TP}}{(2\ast \text{TP}+\text{FN}+\text{FP})}$$

52 $$\text{RMSE}= \sqrt{\frac{1}{\text{N}}\sum_{\text{s}=1}^{\text{N}}{\left({\text{b}}_{\text{s}}-\text{f}\left({\text{a}}_{\text{s}}\right)\right)}^{2}}$$

High-speed filters are used to preprocess the raw ECG dataset that is provided. The ECG dataset, which is divided into sub-classes, is used to extract features and classify them using a variety of techniques. Following the completion of all the preparatory processes, the neural network model classifies the signals to forecast cardiac problems by evaluating the signal patterns using several ECG features. Additionally, the suggested model looks into the error rate, which is significantly lower than in previous methods.

**Figure 8:**
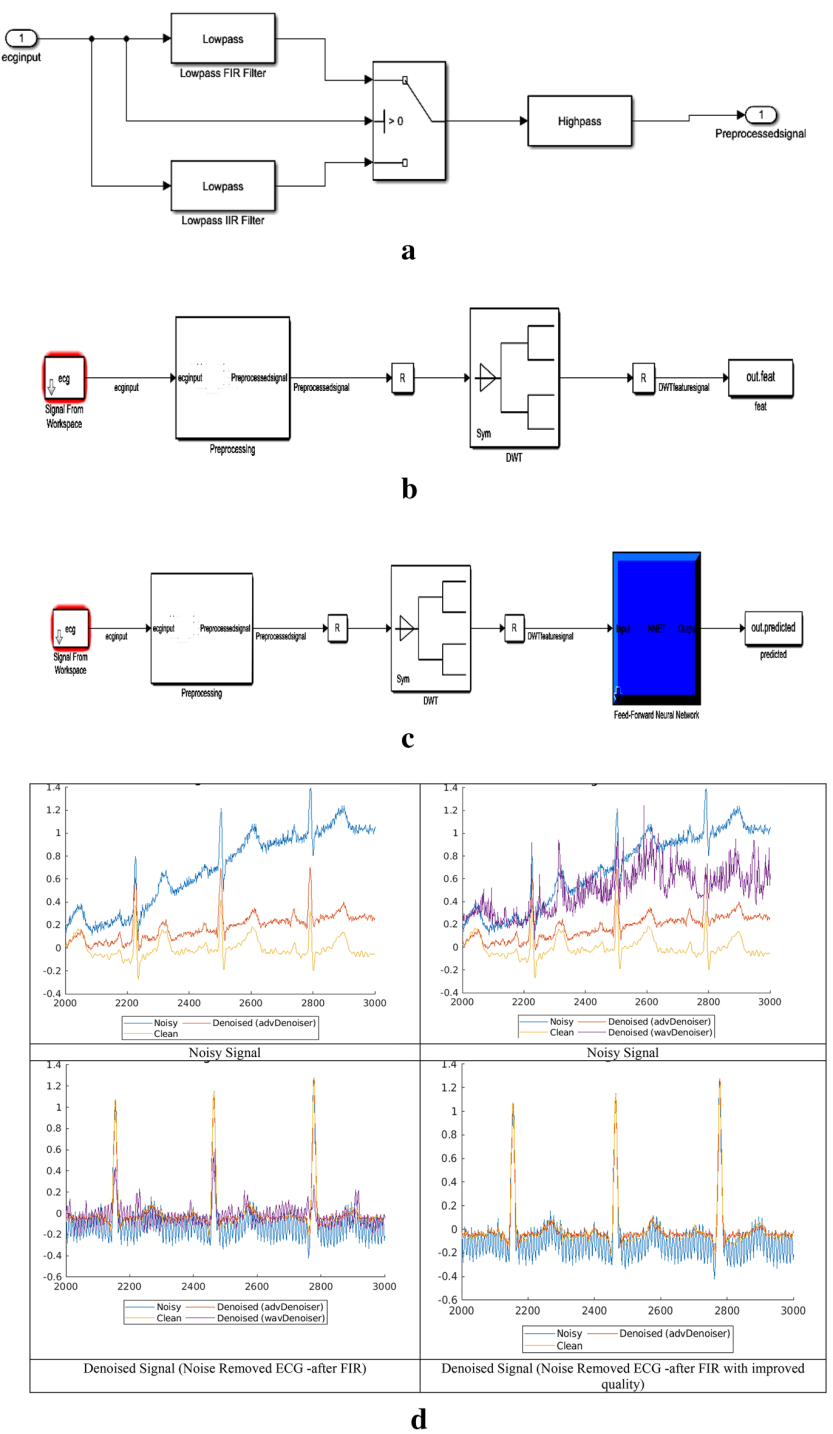
(**a**) Preprocessed parameter using the LPF IIR–FIR filters on selection. (**b**) Training model using the SWT methods from the signals extracted and filtered by the pre-processing block. (**c**) Testing model by the re-constructed neural network. (**d**) Reconstructed ECG Signal after removing noise and improvised the Quality using MFIRs.

Table 4 presents a comparison of the expected model using various methods for ECG-signal based prediction. Figure 8a illustrates the proposed model of modified FIR. Here it is controlled by the bidirectional switch for selecting the FIR and IIR filters. The signals are extracted through the models of DB and its preprocessed by the filter of FIR or IIR. Figure 8b shows the Feature extraction method using DWT method and its processed DWT blocks. Similarly, Fig. 8c shows the testing environment and the corresponding results are generated using MAT lab waveform generator tool and its visualized in Fig. 8d. The obtained ECG signals with the noises are producing the poor accuracy. Hence the computation blocks are modified to remove the noise and increase the accuracy level. Therefore, the ML based prediction improves the HRV diagnosis and promised to have good model performance. Accuracy, precision, recall, F1-score, and error rate are the measures. The predicted HNN has a 92.3% accuracy rate, with 3.3%, 5.3%, 23.3%, and 24.3% higher than GSNN, ANN, SVM (linear) and SVM (RBF). The precision of the anticipated model is 93.4% which is comparatively lesser than GSNN and ANN and higher than SVM (linear and RBF).

The suggested HNN has a recall of 98.5%, which is 9.5%, 30.5%, 39.5%, and 38.5% greater than previous methods such as GSNN, ANN, SVM (linear) and SVM (RBF) respectively.

**Figure 9:**
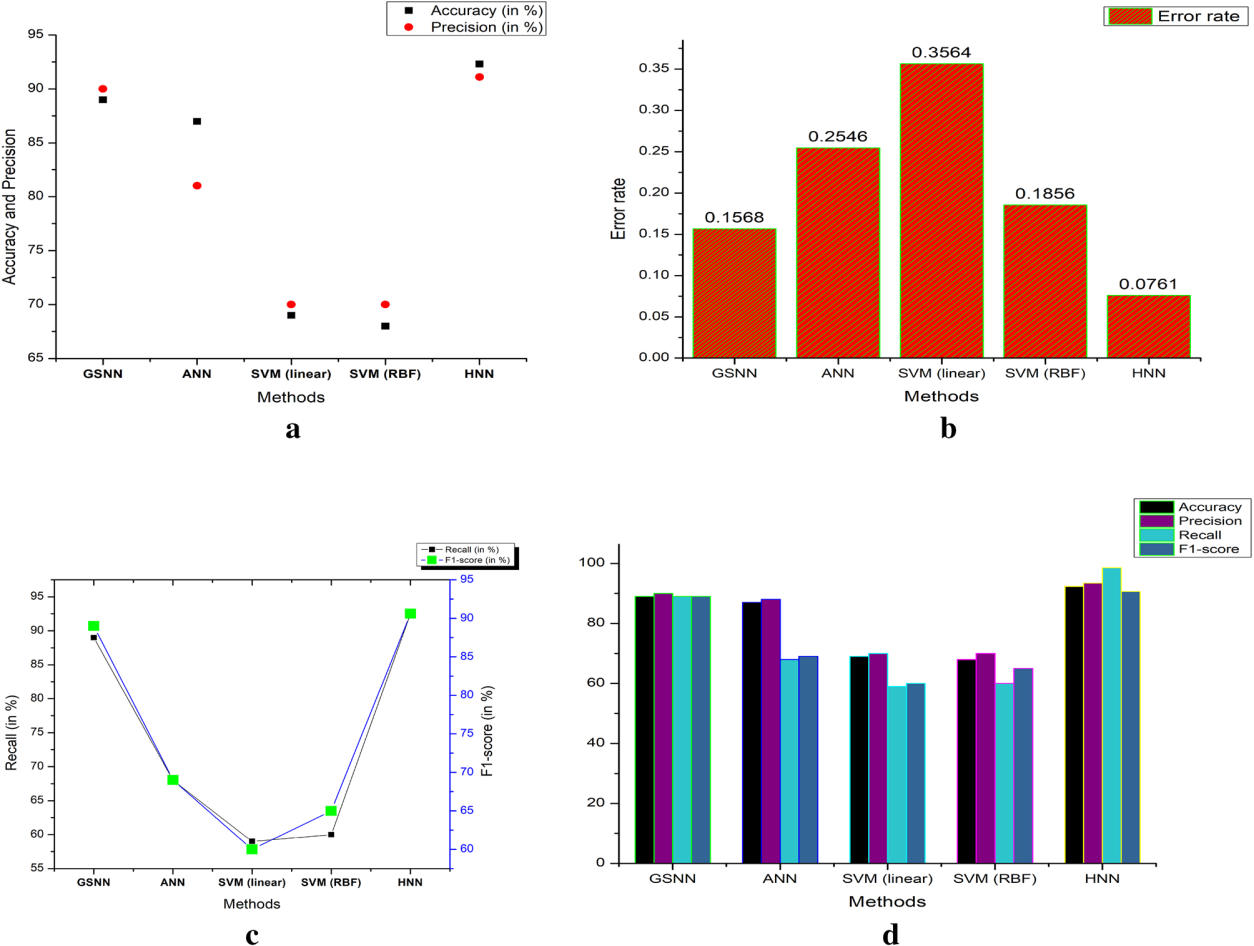
(**a**) Comparative results on accuracy, precision. (**a**) Comparative results on error rate. (**c**) Comparative results on recall and F1 score on various methods vs proposed method. (**d**) Comparative results on accuracy, precision, recall and F1 score on various methods.

The predicted model's F1 score is 90.6%, greater than the other models by 1.6%, 21.6%, 24.6%, and 25.6% methods such as GSNN, ANN, SVM (linear) and SVM (RBF) respectively. Based on these measurements, even if the error rate is significantly lower (0.0761) than with other methods, it is demonstrated that the pre-processing filters effectively reduce noise and produce better results during the prediction process. The lowest error rate assures the models high performance on classifying the ECG by the HRV. The various methods of DL, especially by the CNN, LSTM, auto encoders and Bi-LSTM methods are explained in^34–39^. This DL methods accuracy is improved lot and its in the range of 90% to 99% and even 100% is possible on the ideal and normalized database. The MIT-BITA has the highest accuracy of 99.99% in Bi-LSTM model with the sparse coders. Although DL has better performance parameters, the possible of error rate is higher than the ML. However, the proposed method has the accuracy of 92.3%, which is still better on the ML with DBSC and CBCR methods. Figure 9 illustrates the performance characteristics of the different models listed in Table 4.

**Figure 10:**
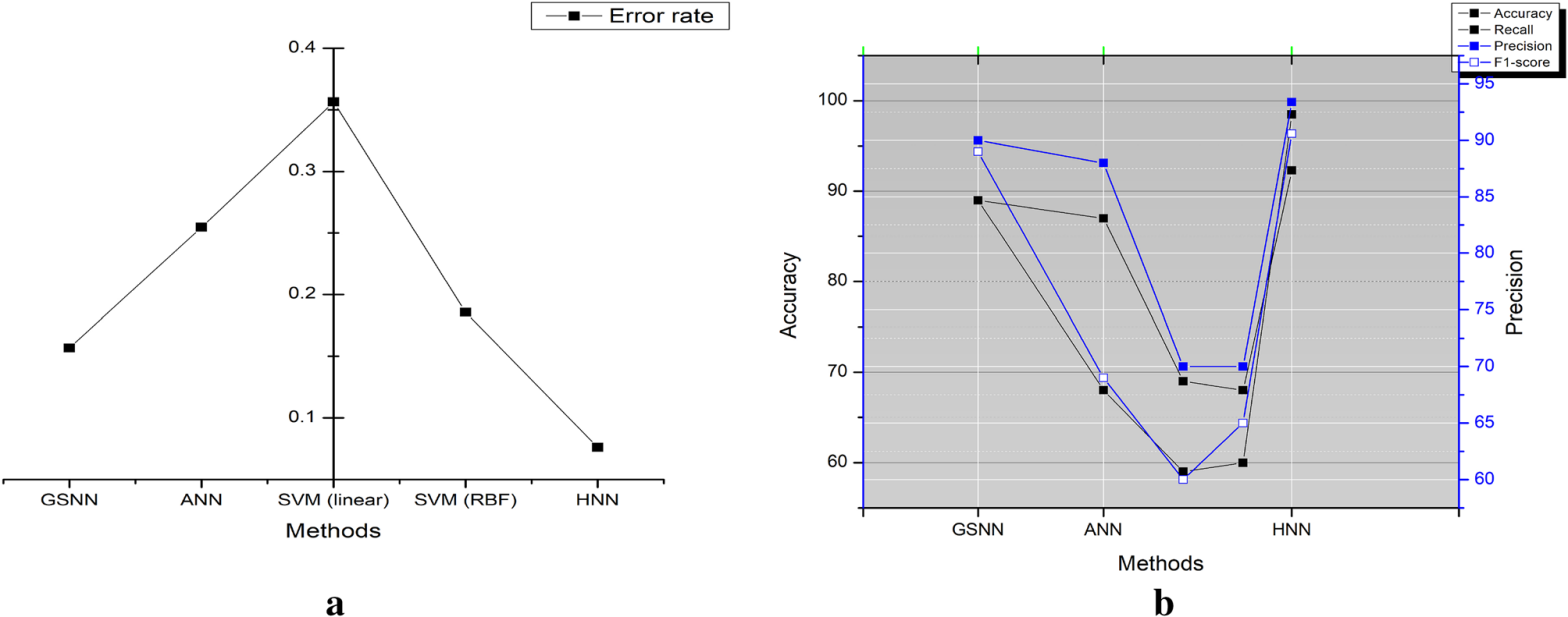
(**a**) Comparative results on error rate of various methods vs existing model. (**b**) Comparative results on accuracy, recall, F1 score and precision of proposed vs various methods.

Figure 9a illustrates the comparative analysis of accuracy and precision, where it is observed that the proposed model has better optimized performance. This is due to the hybrid model preferred for extracting the features and process via HNN models. Figure 9b displays the comparative results on error rate. As the features are selected based on the fusion methods of features. As the pre-processing is performed by MFIR filters, the error is much reducing compare to state of art existing models. For the proposed work the error rate under the fusion method is 0.0761. Figure 9c,d illustrates the overall comparison of the accuracy, precision, F1 score and recall value of the existing system and proposed method. Table 4, shows the overall comparison of the state of art method with the proposed method without hybrid method. The features are never fused here and overshadow the existing models such as GSNN, ANN, SVM (linear) and SVM (RBF). Similarly, Fig. 10a represents the error rate of the different models. The SVM method has the highest error 0.35 and the minimum is 0.07 by the proposed model. The Convergence Region (COR) is represented in the Fig. 10b, which is plotted between the accuracy and precision which are mapped with the recall and F1 score values. In the cased of COR, the performance of proposed methods is better than the other existing models. Table 5 shows the comparative statement of accuracy and precision for the data source on the participants list combination. The accuracy is defined as the ratio of TP to sum of TP, TN, FP and FN as specified in Eqs. (49–52). The precision is computed by the Eq. (49). Table 5 list the DB and the results are separated based on the extracted features. The features considered here are Wavelets, Fiducial, Stats and Correlations. Every DB are run through for the extraction of two discriminative features. The accuracy of all DB is in the range of 83.5% to 92.3%. The minimum accuracy is obtained in PTB DB under the correlation method. The DB are sub sectioned into 15%, 30% and 60% of the subjects of participants. The limitation of executing is unbounded in this analysis instead of various epochs, the HNN are expressed to analyses in the limited participants. The multi-functional library source of python is utilized here. To evaluate the analysis the results shown, the weighted probability is introduced at each stage prior to the execution. The proportion of the analysis are taken at the different portion and at the distinct age groups.

**Table 5:** Data Source and comparative analysis on different participants list for accuracy and precision.

Data source	Extracted features	No of participants (in %)					
		15		30		60	
		Accuracy	Precision	Accuracy	Precision	Accuracy	Precision
MIT-BITA	Discrete wavelets	89.4	80.3	87.6	78.7	86.5	77.7
	Stats	87.7	78.7	85.9	77.2	84.9	76.2
PTB	Correlations	83.5	75.0	81.8	73.5	78.6	70.5
AHA—Physionet	Cross correlations	85.3	76.6	83.6	75.1	80.3	72.0
	Fiducial	84.9	76.4	83.2	74.9	75.7	68.1
EDB	Discrete wavelets	86.8	78.1	85.1	76.6	77.4	69.7
	Stats	87.1	82.4	85.4	80.7	78.5	74.3
St Petersburg DB	Discrete wavelets	89.4	84.6	87.6	82.9	80.6	76.2
	Stats	86.8	79.9	85.1	78.3	79.1	72.8
Proposed-MDB	Discrete wavelets	92.3	90.3	91.0	89.0	90.5	88.5
	Stats	91.7	89.7	90.4	88.4	89.9	87.9

**Figure 11:**
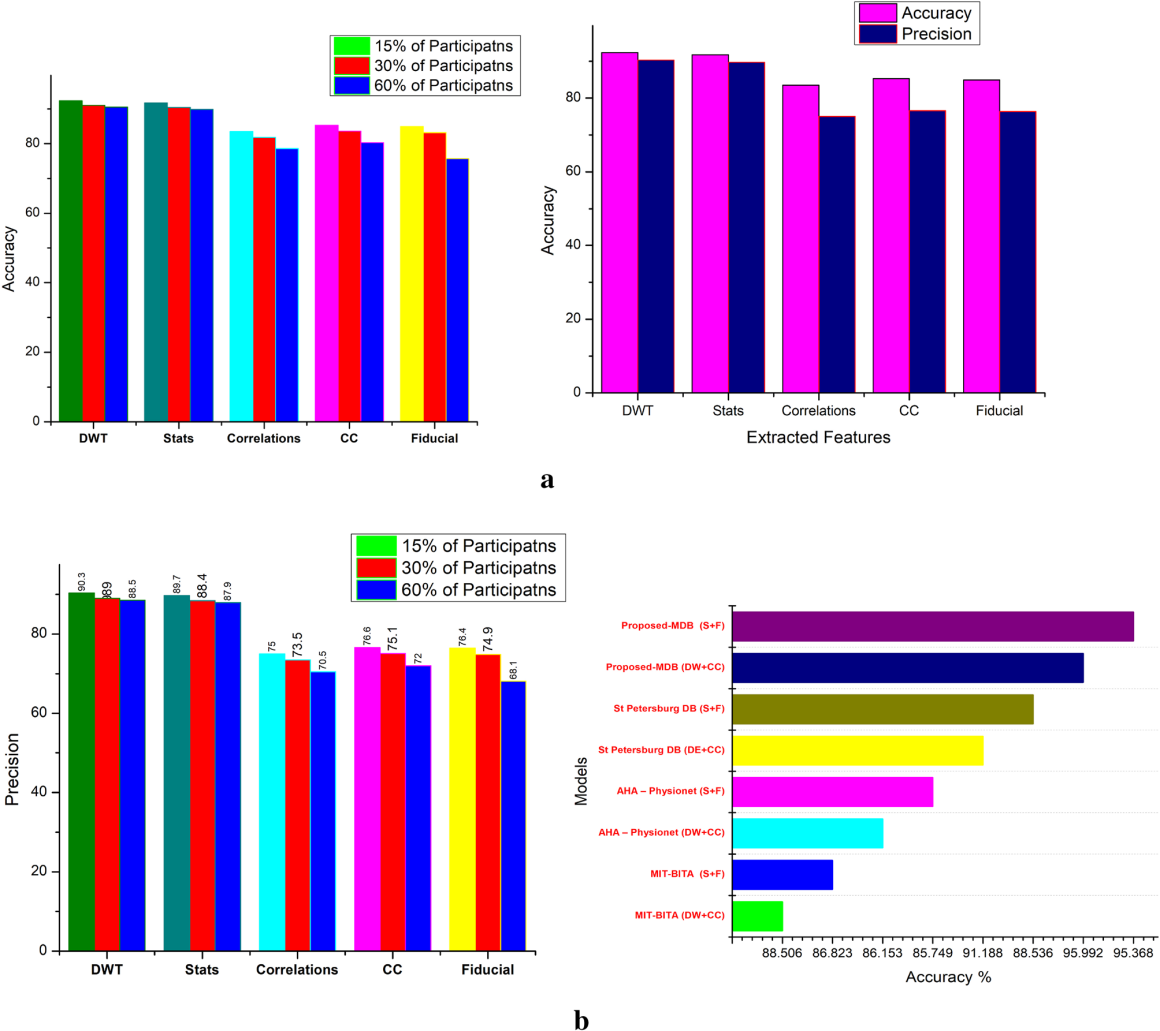
(**a**) Participants database comparison on the features values of accuracy. (**b**) Comparative results on extracted features of various database; *S* statistical, *F* fiducial, *DW* discrete wavelets, *CC* cross correlation based on the segregation of data base.

The precision value is in the range of 75% to 90.3%. In the comparison chart it is noted that the PDB under hybrid architecture has maximized performance of 92.3% for accuracy and 90.3% for the accuracy. Similar to this for statistical feature extractions 91.7% for accuracy and 89.7% for the accuracy. The PMDB in the HNN has better accuracy of more than 10% in PTB, higher than 8% in AHA physionet, 6% improvise compare to EDB 5% higher than the MIT-BIT for 15 subjects. Similar to the accuracy the precision also improved lot. Precision is improved by more than 17% in PTB, higher than 15% in AHA physionet, 13% improvise compare to EDB 11% higher than the MIT-BIT for 15 subjects. Also, for the scale of comparison on 30 subjects and 60 subjects, the accuracy is improved by 10% of maximum (PTB) and 4% of minimum (MIT-BIT). The results shows better performance of the HNN for the optimized dataset under DBSC method of clusters. Figure 11, represents the accuracy, precision variation of the subjects from 15 to 60.

**Figure 12:**
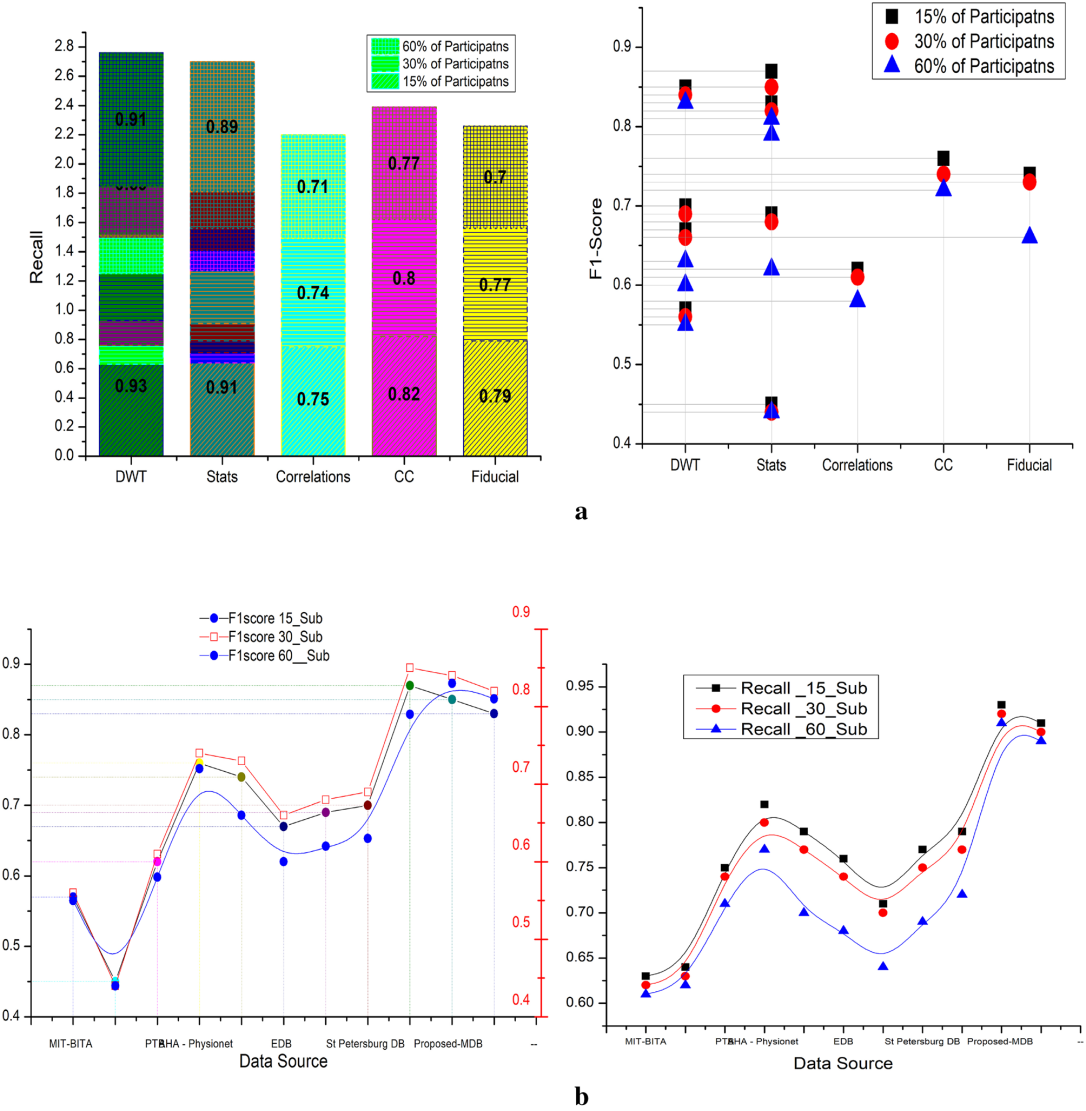
(**a**) Comparative results on extracted features on various database of recall. (**b**) Comparative results on extracted features on various database of F1score.

Table 6 list the other parameter computed from the extracted features. The computed parameters are Recall and F1 score. The metric F1 shows the relation between the accuracy and precision and the highest value for the precision is 0.91. The F1 score shows the harmonic variation with respect to the recall and precision. The MIT-BITA has the average recall and F1 value of 0.62 and 0.56. The PTB has 0.63, 0.44 and AHA has 0.73 and 0.60. For EDB and STB its vary between 0.73 and 0.84 in F1 score and recall values. Among this method PMDB by the Hybrid architecture produces 0.88 as average recall and 0.82 as average precision. As per^35^ the models which has F1 value and recall values closer to 1, will perform as perfect classifier. In percentage the PMDB’s recall score are higher than 29% of MIT-BITA, 17% of MIT-BITA stats, 9% for PTB, 14% of AHB physionet, 23% of STB, and 16% of STB stats. Similarly, the F1 score of PMD are higher than 47% of MIT-BITA, 28% of MIT-BITA stats, 16% for PTB, 24% of AHB physionet, 13% of STB, and 20% of STB stats. The recall states that how many times the classifier were hitting the true prediction. Figure 12a,b illustrates the parameter relation between the DB and the corresponding values of recall and f1 score.

**Table 6:** Available data base with the computed recall, F1 score on different participants.

Data source	Extracted features	No of participants (in %)					
		15		30		60	
		Recall	F1 score	Recall	F1 score	Recall	F1 score
MIT-BITA	Discrete wavelets	0.63	0.57	0.62	0.56	0.61	0.55
	Stats	0.64	0.45	0.63	0.44	0.62	0.44
PTB	Correlations	0.75	0.62	0.74	0.61	0.71	0.58
AHA—Physionet	Cross correlations	0.82	0.76	0.80	0.74	0.77	0.72
	Fiducial	0.79	0.74	0.77	0.73	0.70	0.66
EDB	Discrete wavelets	0.76	0.67	0.74	0.66	0.68	0.60
	Stats	0.71	0.69	0.70	0.68	0.64	0.62
St Petersburg DB	Discrete wavelets	0.77	0.70	0.75	0.69	0.69	0.63
	Stats	0.79	0.87	0.77	0.85	0.72	0.79
Proposed-MDB	Discrete wavelets	0.93	0.85	0.92	0.84	0.91	0.83
	Stats	0.91	0.83	0.90	0.82	0.89	0.81

Table 7, shows the results obtained for the hybrid to enhance the quality of the classifications. The features are combined together to represent the hybrid DB. The hybrid DB are the combination of different features such as Discrete wavelets, Cross correlations, Fiducial and Cross Correlations. As the models are concern the combinations are wavelet with correlations and stats with fiducial is possible. Other combinations of DB based features does not have adequate response. Therefore, these two combinations are chosen to the run the process. The similar pattern of analysis is derived as mentioned in Table 6. The average improvement is considered for all the 60% of participants.

**Table 7:** Hybrid data base with the computed accuracy and precision on different participants.

Data source	Extracted features	No of participants (in %)					
		15		30		60	
		Accuracy	Precision	Accuracy	Precision	Accuracy	Precision
MIT-BITA	Discrete wavelets + cross correlations	88.51	79.46	85.68	76.93	86.28	77.46
	Stats + fiducial	86.82	77.95	84.06	75.46	84.64	75.99
AHA—Physionet	Discrete wavelets + cross correlations	86.15	77.35	85.27	76.55	80.05	71.87
	Stats + fiducial	85.75	77.17	84.87	76.38	75.52	67.97
St Petersburg DB	Discrete wavelets + cross correlations	91.19	86.25	88.49	83.69	84.71	80.12
	Stats + fiducial	88.54	81.45	85.91	79.04	83.14	76.49
Proposed-MDB	Discrete wavelets + cross correlations	95.99	93.88	93.28	91.23	92.27	90.24
	Stats + fiducial	95.37	93.27	92.68	90.64	91.67	89.65

The average accuracy of MIT-BITA is 86.82 for DW + CC and 85.17 for ST + F, for AHA it is 83.82 for DW + CC and 82.82 for ST + F, for STB it is 88.13 for DW + CC and 85.86 for ST + F. The PMDB has the accuracy of 93.85 for DW + CC and 83.24 for ST + F. The average precision of MIT-BITA is 77.95 for DW + CC and 76.477 for ST + F, for AHA it is 75.26 for DW + CC and 73.84 for ST + F, for STB it is 83.35 for DW + CC and 79.00 for ST + F.

**Figure 13:**
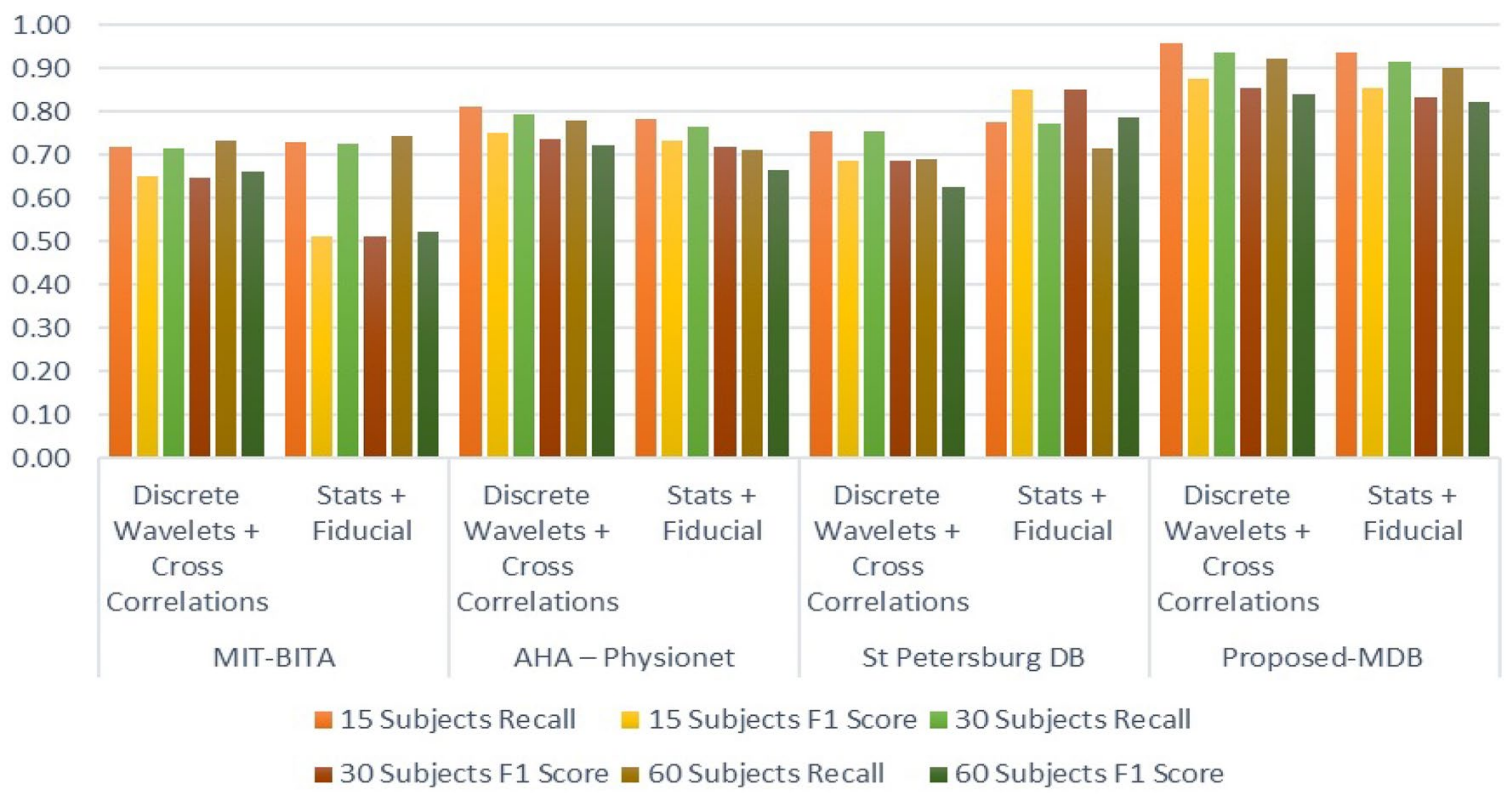
Comparative results on hybrid features on various database of F1score and Recall.

The PMDB has the accuracy of 91.78 for DW + CC and 91.19 for ST + F. Table 8 shows the comparative experimental results of hybrid DB and the mixed feature extraction methods. The average recall of MIT-BITA is 0.72 for DW + CC and 0.73 for ST + F, for AHA it is 0.80 for DW + CC and 0.75 for ST + F, for STB it is 0.73 for DW + CC and 0.75 for ST + F. The PMDB has the accuracy of 0.94 for DW + CC and 0.92 for ST + F. The average F1 score of MIT-BITA is 0.65 for DW + CC and 0.52 for ST + F, for AHA it is 0.74 for DW + CC and 0.71 for ST + F, for STB it is 0.67 for DW + CC and 0.72 for ST + F. The PMDB has the accuracy of 0.86 for DW + CC and 0.84 for ST + F. Figure 13 illustrates the comparative results of hybrid feature extracted DB utilized in HNN with the existing methods.

**Table 8:** Hybrid data base with the computed recall and F1 score on different participants.

Data source	Extracted features	No of participants (in %)					
		15		30		60	
		Recall	F1 score	Recall	F1 score	Recall	F1 score
MIT-BITA	Discrete wavelets + cross correlations	0.72	0.65	0.72	0.65	0.73	0.66
	Stats + fiducial	0.73	0.51	0.73	0.51	0.74	0.52
AHA—Physionet	Discrete wavelets + cross correlations	0.81	0.75	0.80	0.74	0.78	0.72
	Stats + fiducial	0.78	0.73	0.77	0.72	0.71	0.67
St Petersburg DB	Discrete wavelets + cross correlations	0.75	0.69	0.75	0.69	0.69	0.63
	Stats + fiducial	0.77	0.72	0.77	0.85	0.74	0.79
Proposed-MDB	Discrete wavelets + cross correlations	0.96	0.88	0.94	0.85	0.92	0.84
	Stats + fiducial	0.94	0.85	0.92	0.83	0.90	0.82

Here, the accuracy of the model in hybrid is improved between 7 ± 10% and the precision is improved between 9 ± 16%. Also, this mixed hybrid method shows the notable improvement in other parameters such as recall and F1 score. The parameters are improved between 15 ± 20%.

**Figure 14:**
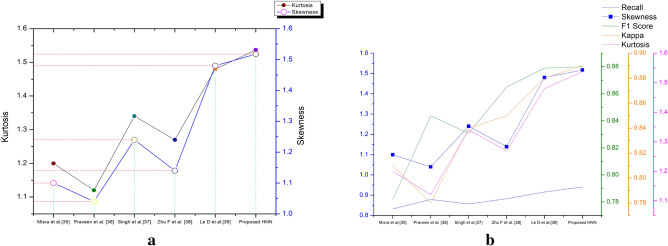
(**a**) Existing method vs proposed method on kurtosis and skewness. (**b**) Existing method Vs proposed method recall, Kappa and F1 Score.

Table 9 discuss the certain existing methods which were used for ECG classification. Mistra et al.^35^ discuss the parametric approach on the classification of the ECG signal. In this paper, cubic spline are generated from the ECG DB and the classification is performed by the SVM (Linear), Tree classifier and CN2 rule. The ML models focus on classifying the abnormalities of HRV in sinus rhythms. Figure 14a,b depicts the kurtosis, skewness and recall, Kappa and F1 Score of the proposed system and its comparison with the existing systems.

**Table 9:** Comparative results on proposed method with the various existing methods.

Existing method	Extraction type/method	Classifier	Accuracy (in %)	Precision (in %)	Recall	F1 score	Kappa	Kurtosis	Skewness
Misra et al.^35^	SVM	C + ST	91.24	90.82	0.83	0.78	0.81	1.20	1.10
Praveen et al.^36^	Spatial temporal	ST	93.81	91.49	0.88	0.84	0.78	1.12	1.04
Singh et al.^37^	RCNN	DW	94.15	91.80	0.86	0.83	0.84	1.34	1.24
Zhu F et al.^38^	Evolutionary	Fi + AC	93.99	91.40	0.88	0.86	0.85	1.27	1.14
Le D et al.^39^	Deep features	DW	97.40	94.40	0.92	0.88	0.88	1.48	1.39
Proposed HNN	Hybrid features	DW + CC + S	95.99	93.88	0.94	0.88	0.89	1.54	1.52

*DW* discrete wavelet transforms, *CC* cross correlations, *S* stats, *ST* statistics, *AC* auto correlation.

This systems performance is limited due to un-arranged preprocessing steps. In another method, the Auto encoder with Extreme Gradient boosting (XGB) is combined to create the hybrid model. This methodology proposed by Praveen et al.^36^ and it refers the spatial temporal features for the classifications. This method has the 99.99% of accuracy on ideal database. Sign et al.^37^ proposed method for classification of ECG using RNN. Her the PTB and MIT- BITA data bases are used in sample testing and training phases. The grey wolf optimization method is used here for the high accurate classification; however, the failure of the DB causes the systems performance as lower and the hybrid technology is implemented. Similar to this method Zhu et al.^38^ and Le et al.^39^ proposed method for classification of ECG with the evolutionary and supervised methods. Both methods support several leads. These methods are contrastive on learning with respect to the transformations and the algorithms to conclude the HRV diseases. The comparison results shows that the accuracy of the proposed model is 95.99%. Its lower than the model exhibited in^39^ which are based on the deep features. Also, other methods have the accuracy between 91.24 in^35^ and 94.15 in^37^. Based on utilization of DB under the composite features and classifier shows the better results.

**Figure 15:**
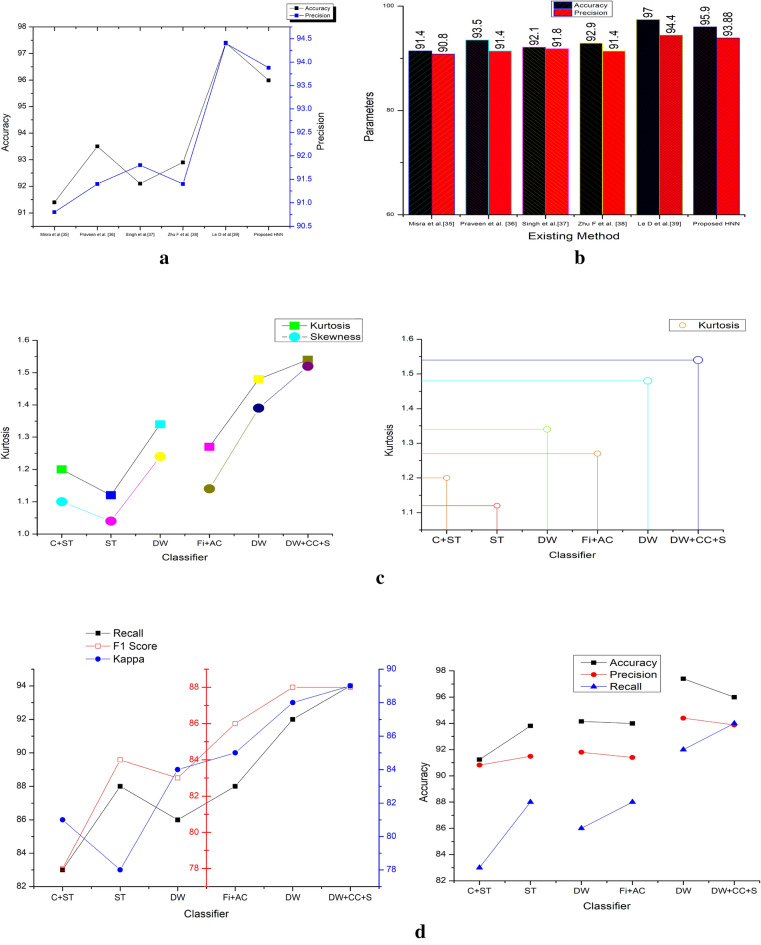
(**a**) Existing method vs proposed method on accuracy and precision. (**b**) Existing method vs proposed hybrid method on accuracy. (**c**) Existing method vs proposed hybrid method on kurtosis and skewness. (**d**) Existing method vs proposed hybrid method on accuracy, precision, F1 score and recall.

The precision of the proposed method is 93.88% which is slightly lower than the deep feature model proposed in^39^, whereas other models have the recall divergence of 90.82 ± 91.8. Also, extended analysis of other parameters also improved in the proposed method. The recall of the proposed method is 0.94 and the F1 score is 0.88. The band of deviation is 0.83 ± 0.92 for recall and 0.78 ± 0.92 for F1 score. The kapa value is computed for the models and the value are between − 1 and + 1. For the proposed model the Kappa is 0.89, which is highly recommend the HNN model is more effective and the predicted results are in-line with the actual results. The skewness and the kurtosis indicate the extreme symmetry and asymmetry distribution of the data with long or light tail representation on the DBSC. Here, kurtosis is 1.54 and the skewness is 1.52, which is falls on the platykurtic region. This shows the most of the data are relevant and distributed with the high proximate value such as mean, std deviations. Figure 15a depicts the existing method vs proposed method on accuracy and Precision and Fig. 15b illustrates the Existing method vs proposed hybrid method on accuracy.

As the overall experiment analysis is concern, the notable improvement is observed in all the aspects of ML parameters in this HNN. The accuracy is switched to 95.99%, precision 93.88%, recall is 0.94, F1 score is 0.88, Kappa is 0.89, kurtosis is 1.54 and skewness is 1.52. The HNN model gradually improvise the performance from 91.22% of 15 subject participant to 95.99% of mixed hybrid NN model.

**Figure 16:**
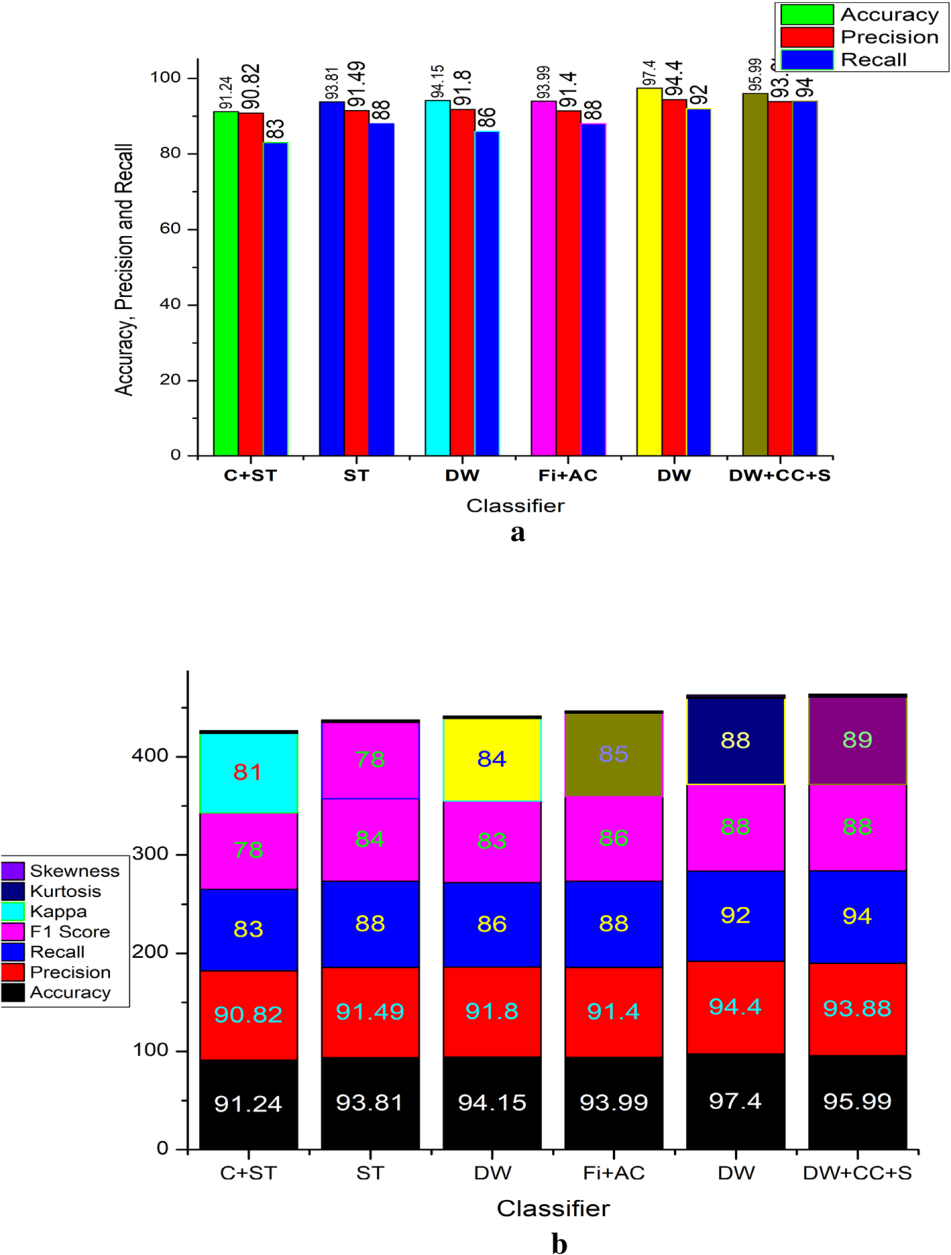
(**a**) Overall comparison of proposed method vs existing method, analysis based on ML parameters and hybrid models of features. (**b**) Overall comparison of proposed method vs existing method, analysis based on ML parameters and hybrid models of features.

Figure 16 shows the overall comparison of the HNN based proposed method. It includes all the possible features extracted from the ECG signals. All the ECG are publicly available. The noises are removed using the MFIR and supports the system to improve the accuracy of predictions. Figure 15c,d visualized the comparative results of kappa and kurtosis parameters are combined with the existing methods. Incorporating kappa and kurtosis parameters can significantly enhance the performance of hybrid neural networks in ECG classification by capturing critical statistical characteristics of ECG signals. Kappa measures the shape of the data distribution and provides insights into the general morphology of ECG waveforms. This parameter helps in distinguishing between normal and abnormal ECG patterns by quantifying variations in the waveform's shape. This reducing the influence of noise and artifacts. Kappa can identify subtle deviations in ECG signals that are indicative of different cardiac conditions, enriching the feature set used in the classification process. When integrated into hybrid neural networks, kappa values augment the feature vector by adding valuable morphological information, complementing time and frequency domain features. Also, improving the network's ability to model and recognize various ECG patterns.

Kurtosis, quantifies the peak or flatness of a signal's distribution, providing a measure of the data distribution deviates from normal to abnormal conditions. High kurtosis values indicate the presence of sharp spikes or outliers, which can be associated with abnormal cardiac events, while low kurtosis suggests a flatter distribution that might indicate motion artifacts or baseline wander. By including kurtosis in the feature set, hybrid neural networks can better detect and classify abnormal ECG patterns. The kurtosis highlights significant deviations that are often crucial in identifying pathological conditions. This helps the network to distinguish between different forms of cardiac anomalies more effectively. Therefore, the integration of kappa and kurtosis parameters into hybrid neural networks provides a more nuanced representation of ECG signals, leading to richer and more informative feature sets. This enhanced representation improves the accuracy of ECG classification by allowing the network to leverage both statistical and morphological features. The result is a more robust and reliable model for detecting a wide range of heart conditions, enhancing the clinical utility of ECG monitoring systems.

Hence the classification of ECG om HRV is improved lot and the preprocessing is filtered by the designed FIR and IIR filter specified in the sections "Proposed work" and “Classification using neural network and hybrid tuned parameters”. The application of ECG signal classification using hybrid machine learning models significantly enhances cardiac care, offering numerous benefits in clinical and remote settings. One critical application is arrhythmia detection, where timely identification of irregular heartbeats can prevent severe events such as strokes or heart attacks. Hybrid models for classification improves the accuracy and reliability of these detections. Apart from this, it can be used in (i) personalized medicine, (ii) health monitoring-ECG, (iii) clinical research-result, (iv) personalization assistance, (iv) forecast future cardiac diseases in timely manner.

## Conclusion

Modern wearable ECG-based real-time monitoring devices require high speed and low power filtering for the prediction model to function during real-time processing. Designing a filter requires a high-performance, low-power filter unit, which is extensively covered in this work. The predicted filtering model's performance is examined and contrasted with several alternative methods using a range of prediction measures. Ideas for resolving the problems with the general approaches are stimulated by the use of HNN for classification, wavelet transforms for feature analysis, and FIR and IIR based filter design. The most popular method of filtering is using digital filters with windows since they are faster, more linear, and simpler to use. High-speed filters are so created, and they can be used in portable devices to help the human community. With the existing models and experiment results shows better performance. The experimental results depicts that the accuracy of the anticipated Hybrid Neural Network (HNN) model reached 92.3%, surpassing other models such as Generalized Sequential Neural Networks (GSNN), Artificial Neural Networks (ANN), Support Vector Machine with linear kernel (SVM linear), and Support Vector Machine with Radial Basis Function kernel (SVM RBF) by margins of 3.3%, 5.3%, 23.3%, and 24.3%, respectively. While the precision of the anticipated model stood at 73.4%, it was slightly lower than GSNN and ANN but higher than both SVM linear and SVM -RBF. The HNN with various features are incorporated to improve the ECG classification. The accuracy of the HNN is switched to 95.99%, precision 93.88%, recall is 0.94, F1 score is 0.88, Kappa is 0.89, kurtosis is 1.54 and skewness is 1.52. These parameters are higher than recently developed models whose algorithms and methods accuracy is more than 90%.

### Summary

- The Electrocardiogram (ECG) records are crucial for predicting heart diseases and evaluating patients' health conditions.
- In ECG signal denoising, digital filters like Infinite Impulse Response (IIR) and Finite Impulse Response (FIR) are commonly used, with FIR filters preferred for their higher-order performance and stability over IIR filters, especially in real-time applications.
- Features are analyzed using techniques like wavelet transform, and the extracted features are inputted into classifier models.
- Various Performance metrics such as precision, recall, accuracy and F-measure are compared and evaluated against other approaches.
- For instance, in a recent study, the accuracy of the anticipated Hybrid Neural Network (HNN) model reached 92.3%, surpassing other models such as Generalized Sequential Neural Networks (GSNN), Artificial Neural Networks (ANN), Support Vector Machine with linear kernel (SVM linear), and Support Vector Machine with Radial Basis Function kernel (SVM RBF) by margins of 3.3%, 5.3%, 23.3%, and 24.3%, respectively.
- While the precision of the anticipated model stood at 73.4%, it was slightly lower than GSNN and ANN but higher than both SVM linear and SVM -RBF.
- The HNN with various features are incorporated to improve the ECG classification. The accuracy of the HNN is switched to 95.99%, precision 93.88%, recall is 0.94, F1 score is 0.88, Kappa is 0.89, kurtosis is 1.54 and skewness is 1.52.
- These parameters are higher than recently developed models whose algorithms and methods accuracy is more than 90%.

## Data Availability

In this article, publicly available data base is used (MIT-BIH Arrhythmia Database v1.0.0 (physionet.org)) and relevant database git hub repository is available in Dineshkumarjr/ECG (github.com).
